# The Role of Tumor Microenvironment in Chemoresistance: 3D Extracellular Matrices as Accomplices

**DOI:** 10.3390/ijms19102861

**Published:** 2018-09-20

**Authors:** Dimakatso Alice Senthebane, Tina Jonker, Arielle Rowe, Nicholas Ekow Thomford, Daniella Munro, Collet Dandara, Ambroise Wonkam, Dhirendra Govender, Bridget Calder, Nelson C. Soares, Jonathan M. Blackburn, M. Iqbal Parker, Kevin Dzobo

**Affiliations:** 1 Division of Medical Biochemistry and Institute of Infectious Disease and Molecular Medicine, Faculty of Health Sciences, University of Cape Town, Anzio Road, Observatory, Cape Town 7925, South Africa; SNTDIM001@myuct.ac.za (D.A.S.); tyjonker@gmail.com (T.J.); iqbal.parker@uct.ac.za (M.I.P.); 2 International Centre for Genetic Engineering and Biotechnology (ICGEB), Cape Town Component, Wernher and Beit Building (South), UCT Campus, Anzio Road, Observatory, Cape Town 7925, South Africa; arielle.rowe@icgeb.org; 3 Pharmacogenetics Research Group, Division of Human Genetics, Department of Pathology and Institute of Infectious Diseases and Molecular Medicine, Faculty of Health Sciences, University of Cape Town, Anzio Road, Observatory, Cape Town 7925, South Africa; Nicholas.thomford@uct.ac.za (N.E.T.); MNRDAN002@myuct.ac.za (D.M.); collet.dandara@uct.ac.za (C.D.); Ambroise.wonkam@uct.ac.za (A.W.); 4 Division of Anatomical Pathology, Faculty of Health Sciences, University of Cape Town, NHLS-Groote Schuur Hospital, Cape Town 7925, South Africa; dhiren.govender@uct.ac.za; 5 Division of Chemical and Systems Biology, Department of Integrative Biomedical Sciences, Faculty of Health Sciences, Institute of Infectious Disease and Molecular Medicine, University of Cape Town, Cape Town 7925, South Africa; bridget.calder@uct.ac.za (B.C.); nelson.dacruzsoares@uct.ac.za (N.C.S.); jonathan.blackburn@uct.ac.za (J.M.B.)

**Keywords:** esophageal cancer, 3D extracellular matrix, stroma, type I collagen, fibronectin, chemoresistance, signaling cascade, targeted therapy

## Abstract

Background: The functional interplay between tumor cells and their adjacent stroma has been suggested to play crucial roles in the initiation and progression of tumors and the effectiveness of chemotherapy. The extracellular matrix (ECM), a complex network of extracellular proteins, provides both physical and chemicals cues necessary for cell proliferation, survival, and migration. Understanding how ECM composition and biomechanical properties affect cancer progression and response to chemotherapeutic drugs is vital to the development of targeted treatments. Methods: 3D cell-derived-ECMs and esophageal cancer cell lines were used as a model to investigate the effect of ECM proteins on esophageal cancer cell lines response to chemotherapeutics. Immunohistochemical and qRT-PCR evaluation of ECM proteins and integrin gene expression was done on clinical esophageal squamous cell carcinoma biopsies. Esophageal cancer cell lines (WHCO1, WHCO5, WHCO6, KYSE180, KYSE 450 and KYSE 520) were cultured on decellularised ECMs (fibroblasts-derived ECM; cancer cell-derived ECM; combinatorial-ECM) and treated with 0.1% Dimethyl sulfoxide (DMSO), 4.2 µM cisplatin, 3.5 µM 5-fluorouracil and 2.5 µM epirubicin for 24 h. Cell proliferation, cell cycle progression, colony formation, apoptosis, migration and activation of signaling pathways were used as our study endpoints. Results: The expression of collagens, fibronectin and laminins was significantly increased in esophageal squamous cell carcinomas (ESCC) tumor samples compared to the corresponding normal tissue. Decellularised ECMs abrogated the effect of drugs on cancer cell cycling, proliferation and reduced drug induced apoptosis by 20–60% that of those plated on plastic. The mitogen-activated protein kinase-extracellular signal-regulated kinase (MEK-ERK) and phosphoinositide 3-kinase-protein kinase B (PI3K/Akt) signaling pathways were upregulated in the presence of the ECMs. Furthermore, our data show that concomitant addition of chemotherapeutic drugs and the use of collagen- and fibronectin-deficient ECMs through siRNA inhibition synergistically increased cancer cell sensitivity to drugs by 30–50%, and reduced colony formation and cancer cell migration. Conclusion: Our study shows that ECM proteins play a key role in the response of cancer cells to chemotherapy and suggest that targeting ECM proteins can be an effective therapeutic strategy against chemoresistant tumors.

## 1. Introduction

Great interest has been generated in developing microphysiological systems that can best mimic normal and pathological human conditions in vitro. Most drug discovery assays are performed using in vitro models that do not recapitulate the in vivo tumor microenvironment present during tumor growth, development and treatment [1,2,3,4,5,6]. The lack of good in vitro tumor models limits our understanding of the functional interplay between tumor cells and the tumor microenvironment [7,8,9,10]. The dynamic full in vivo biological repertoire of the tumor microenvironment include many cells such as fibroblasts, endothelial cells, immune cells and the ECM [7,8,9,10,11,12,13,14,15,16]. This ultimately leads to incorrect and misleading claims when it comes to the efficacy of drug candidates. Such scenarios can be avoided by employing in vitro models that better recapitulate the in vivo tumor microenvironment [3,17,18,19,20,21,22,23]. One important constituent of the tumor microenvironment is the extracellular matrix, a meshwork of proteins and glycosaminoglycans [3,17,18,20]. The ECM provides both mechanical and biochemical support for cellular adhesion and migration and acts as a conduit for extracellular cues via its interaction with cell surface receptors. It is known to sequester growth factors and cytokines and these will affect cellular growth and signaling [24,25,26,27,28,29,30]. Thus the ECM is the “theatre” where most cues or signals from diverse sources are integrated into a “specific” message that is relayed to cells. The ECM is synthesised mostly by fibroblasts with the contribution of other cells such as mesenchymal stem cells, macrophages and endothelial cells [31,32,33,34,35,36]. Fibroblasts are the main cellular components of tumor stroma [37,38,39,40,41,42,43,44]. Activated or transformed fibroblasts have a high proliferation rate and generate huge amounts of extracellular matrix [38,39,45,46,47,48,49].

Esophageal cancer is one of the most highly malignant neoplasms and can be classified into two main subtypes, esophageal adenocarcinomas and esophageal squamous cell carcinomas (ESCC), with the majority of deaths from ESCC occurring in developing countries [50,51,52]. ESCC is the third most common cancer in South African men [53,54]. Although promising progress has been attained in treating esophageal cancer, it responds poorly to chemotherapeutic drugs and the mortality still remains high [51,55,56,57,58,59]. Surgery, chemotherapy and radiotherapy are the most widely-used treatment methods but about half of advanced esophageal cancer cases result in recurrence and patients normally succumb to resistant disease [60,61,62,63,64]. There is a lack of understanding of the mechanisms driving the initiation, progression and the occurrence of refractory disease. Besides the gradual accumulation over time of genetic mutations in epithelial cells due to carcinogen exposure, the initiation, progression and response to chemotherapy of many tumors including esophageal cancer depends on the interplay between the stroma and tumor cells. Recent data suggest that the development of chemoresistant disease is beyond cancer cell autonomous mechanisms with the tumor microenvironment emerging as a key player in this phenomenon [50,51,52,65,66,67,68,69,70]. Several reports have shown the involvement of stromal fibroblasts in esophageal cancer angiogenesis and differentiation through the release of biomolecules and ECM synthesis [71,72,73,74,75,76,77]. The expression of several ECM proteins has been shown to be upregulated in many tumors [17,78,79,80]. The so-called ‘hardening of the tumor’ is in fact the deposition and crosslinking of thick fibres mainly made up of collagen and fibronectin [80,81,82,83,84,85]. ESCC stroma is fibrotic due to desmoplasia. Huge amounts of the ECM are deposited during ESCC development. Thus the constituents of the ECM can have a lasting effect on cancer cells. Different types of ECMs have been used to study cancer cell-ECM interactions, with most ECM proteins being purified proteins [3,6,7,8,10,16,18,20,86]. Decellularised cell-derived ECMs contain native ECM proteins, and are cost-effective and easily obtainable [3,10,18,20,87,88,89,90]. Fibroblast-derived decellularised ECM would mimic the desmoplastic microenvironment of the ESCC.

This study investigated the effect of three different cell-derived ECMs on the response of esophageal cancer cells to chemotherapeutic drugs. We reproduced the native ECM microenvironment by employing decellularised ECMs synthesised by fibroblasts and cancer cells. Our data show that fibroblast- and cancer cell-derived ECMs contain similar ECM proteins, though in differing amounts. We report that decellularised ECMs, regardless of origin, induce chemoresistance to cisplatin, 5-fluorouracil and epirubicin. Survival pathways such as the MEK-ERK and PI3K-Akt pathways were activated in the presence of decellularised ECMs. Remarkably, we show that the use of type I collagen- and fibronectin-deficient ECMs and drugs have a synergistic negative effect on esophageal cancer cell proliferation, colony formation and migration. These results suggest that components of the tumor microenvironment underlie aspects of chemoresistance, and are therefore potential drug targets.

## 2. Results

### 2.1. ECM Proteins and Matrix Metalloproteases Expression in Clinical Esophageal Squamous Cell Carcinoma Tissues

**Figure 1:**
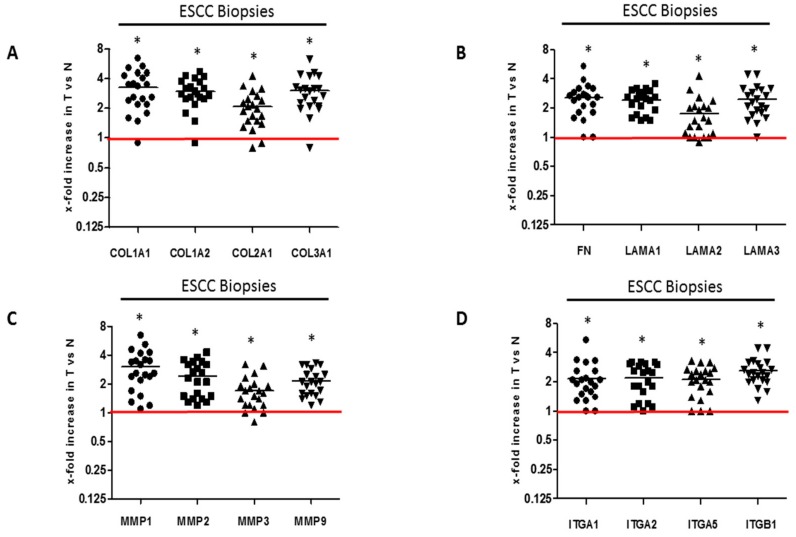
Gene expression profile of ECM proteins and associated proteins in ESCC samples: (**A**) qRT-PCR analysis of collagens mRNA expression in human ESCC samples; (**B**) Fibronectin and laminins mRNA expression in human ESCC samples. (**C**) qRT-PCR analysis of MMPs mRNA expressions in human ESCC samples; (**D**) qRT-PCR analysis of integrins mRNA expression in human ESCC samples. Each ESCC tumor (T) mRNA was quantified relative to the corresponding normal sample (N) from the same patient, which is taken as one. GAPDH is the normaliser. Statistical analysis to determine significance difference of gene expression in tumor versus normal sample was done using a 2-tailed non-parametric Mann-Whitney test. * *p* < 0.05.

Twenty-one biopsy samples were collected from histopathologically-confirmed ESCC patients and were used to evaluate the expression profiles of ECM and associated genes. The clinicopathological characteristics of the 21 ESCC patients are shown in Table 1. The age range of the patient cohort is 30–83 years with a median age of 55 years. Patients were nearly evenly distributed between male and females. Among the patient cohort, with the exception of one patient with a poorly differentiated tumor and four esophageal tumors which were not graded, all other esophageal tumors were moderately differentiated. To determine the importance of ECM proteins in ESCC, we determined the mRNA levels of several ECM proteins in primary esophageal cancer tumor tissue compared to normal tissue. Real time quantitative reverse transcription polymerase chain reaction (qRT-PCR) analysis of RNA extracted from matched ESCC patients’ tumor and adjacent normal tissues, was performed. GAPDH was used as a normaliser. Our statistical analysis of the resultant data show that the expression of collagens, fibronectin and laminins was significantly upregulated in ESCC tumor tissues compared to the corresponding normal tissue (Figure 1A,B). Immunohistochemical staining of tumor and normal biopsy specimens using anti-type I collagen antibody showed significantly upregulated type I collagen in tumor specimens compared to normal biopsy specimens (Supplemental Figure S1A). The source of the ECM within the tumor microenvironment is both cancer cells and stromal cells including cancer-associated fibroblasts. Real time qRT-PCR analysis of RNA from normal fibroblasts (WI38, FG_0_), transformed CT1 fibroblasts and several ESCC cell lines (WHCO1, WHCO5, WHCO6, KYSE 180, KYSE 450, KYSE 520) show that transformed CT1 fibroblasts express significantly higher ECM proteins than normal fibroblasts (Supplemental Figure S1B–D; Supplemental Figure S2A,B). ESCC cell lines express significantly lower ECM proteins, about 30–50%, compared to normal fibroblasts (Supplemental Figure S1B–D; Supplemental Figure S2A,B).

The tumor microenvironment is not constant, with levels of ECM proteins always fluctuating. Changes in the levels of ECM proteins within tumors can be brought about by matrix metalloproteases (MMPs). Among the MMPs, MMP1, MMP2, MMP9 and MT1-MMP produced by both stromal and tumor cells degrade and migrate through the ECM. Our data show significantly upregulated levels of MMP-1, MMP2, MMP3 and MMP9 mRNA in ESCC tumor tissues compared to the adjacent normal tissues (Figure 1C). Increased expression of ECM proteins might also be accompanied by the over-expression of their receptors, which are responsible for relaying extracellular cues between tumor cells and the tumor microenvironment. Indeed, integrins gene expression in the clinical ESCC tumor tissues show significant higher levels of integrin α-1 (ITGA1), ITGA2, ITGA5 and ITGB1 mRNA compared to normal tissue from the same patient (Figure 1D).

### 2.2. Detailed Analysis of Decellularised ECMs Used in the Study

**Figure 2:**
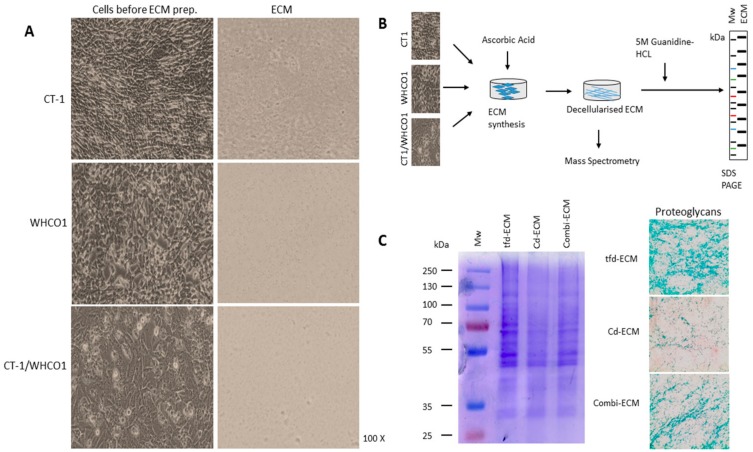
Characterisation of CT1 fibroblasts and WHCO1 cancer cells and their ECMs. (**A**) Phase contrast images of CT1 and WHCO1 cells prior to decellularization (left panel) and phase contrast microscopy of decellularised ECMs (right panel). Scheme of the ECM analysis workflow. Images were taken at 100× magnification. (**B**) Schematic representation of decellularised ECMs synthesis and analysis via SDS PAGE and mass spectrometry. (**C**) Representative image showing SDS PAGE and Coomassie Blue staining of decellularised ECMs (left panel). Representative Alcian Blue staining for proteoglycans in decellularised ECMs. Images were taken at 100× magnification.

Fibroblasts are the major stromal cell type responsible for ECM production and we previously utilised the fibroblast-derived ECM as a model to investigate cancer cell and mesenchymal stem cells interactions [88]. In the context of the tumor, fibroblasts within the tumor or surrounding the tumor are called cancer-associated fibroblasts. We utilised cell-derived 3D culture models involving plating ESCC lines on decellularised ECMs produced by transformed CT1 fibroblasts (fd-ECM), ESCC cell lines (cd-ECM) and a combination of transformed CT1 fibroblasts and WHCO1 cancer cells (combi-ECM) (Figure 2A). Our data show that the transformed CT1 fibroblasts express significantly more upregulated α-smooth muscle actin than normal fibroblasts WI38 and FG0 and show the spindle-shaped morphological features typical of CAFs found within tumors (Supplemental Figure S2C,D). We therefore used transformed CT1 fibroblasts, obtained by transforming WI38 fibroblasts through γ-radiation [91] as our “transformed fibroblasts”. Transformed CT-1 fibroblasts produced an ECM (tfd-ECM) that is much more highly linearized than the ECM produced by WHCO1 cells (cd-ECM) and a mixture of CT-1 fibroblasts and WHCO1 cells (combi-ECM) (Figure 2A, right column). It is important to note that most synthetic and solubilised ECMs used in most experiments such as Matrigel and Fibronectin do not form a linearized ECM. This could have a huge impact on WHCO1 cancer cell response to drugs and probably mimic the in vivo ECM better than these purified ECMs.

In order to study cancer cell-ECM interactions, it is necessary to obtain a detailed composition of the decellularised ECMs. To analyse the composition of the tfd-ECM, cd-ECM and combi-ECM obtained after synthesis, we employed a proteomics pipeline using chromatography combined with tandem mass spectrometry (LC-MS/MS) to identify the peptides and proteins (Figure 2B). Our data showed that all decellularised ECMs generally contain similar ECM proteins and proteoglycans, with obvious differences in the quantities of ECM proteins and proteoglycans identified within tfd-ECM and combi-ECMs compared to cd-ECMs (Figure 2C; Supplemental Figure S3A–C). Mass spectrometric analysis identified well-known ECM proteins within the decellularised ECMs such as collagens, laminins and fibronectin (Table 2). As expected, tfd-ECM and combi-ECM showed higher amounts of ECM proteins such as type I collagen and fibronectin and glycoproteins than cd-ECM (Figure 2C).

### 2.3. Decellularised ECMs Protect WHCO1 Cancer Cells from the Effect of Drugs

We sought to study the proliferation and migration of WHCO1 cancer cells on the underlying decellularised ECMs and to determine how the presence of the ECMs affect the response of WHCO1 cancer cells to chemotherapeutic drugs. Many drugs are used in ESCC chemotherapies, including cisplatin, 5-flurouracil and epirubicin. Cells were treated with 0.1% DMSO (control), cisplatin (*M*_W_ 300.05; CAS 15663-27-1; Sigma Aldrich, Steinheim, Germany), 5-fluorouracil (*M*_W_ 130.08 g/mol; CAS 51-21-8; Sigma Aldrich, Steinheim, Germany), epirubicin (*M*_W_ 579.98 g/mol; CAS 56390-09-1; Sigma Aldrich, Steinheim, Germany) at the indicated concentrations for different time periods. Drugs concentrations used were lower than half of reported and determined IC_50_ values, as determined by the MTT assay, as we were interested in studying the gene response of the WHCO1 cells to these chemotherapeutic drugs and not interested in actually killing the cells. IC_50_ values were measured in WHCO1 cancer cells over 24 h and were determined as the concentration of drugs needed to kill 50% of cells. As shown in Table 3, IC_50_ values for drugs were higher when cancer cells were plated on ECMs compared to plastic. No major morphological changes were observed between WHCO1 cancer cells plated on plastic and those plated on decellularised ECMs, with or without drugs (data not shown).

**Figure 3:**
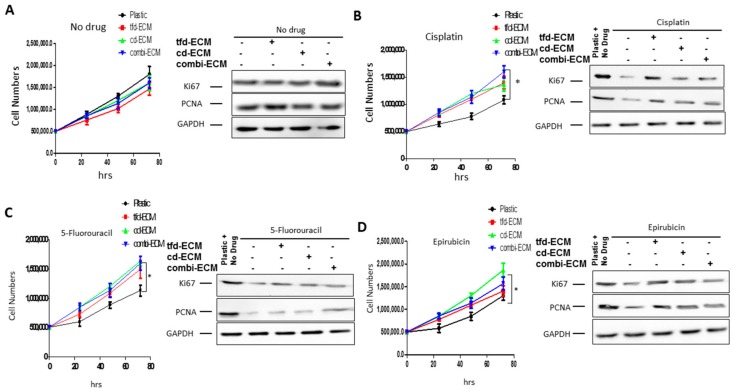
Effect of decellularised ECMs on WHCO1 cancer cell proliferation in response to cisplatin, 5-fluorouracil and epirubicin. WHCO1 cancer cells were cultured on plastic and on ECMs and treated with drugs as indicated for 24 h. Cell counting was done using the Countess Cell Counter. Total proteins (50 µg) were loaded on SDS PAGE gels and immunoblot analysis performed. (**A**) Effect of decellularised ECMs on WHCO1 cancer cell proliferation in the absence of drugs. (**B**) Effect of decellularised ECMs on WHCO1 cancer cell proliferation in response to cisplatin. (**C**) Effect of decellularised ECMs on WHCO1 cancer cell proliferation in response to 5-fluorouracil (**D**) Effect of decellularised ECMs on WHCO1 cancer cell proliferation in response to epirubicin. Data show cell counting (left panel) and immunoblot analysis (right panel). * *p* < 0.05.

With no drug present, there were no significant differences in cell proliferation between WHCO1 cells on plastic and those on decellularised ECMs (Figure 3A). Cisplatin caused a significant decrease in WHCO1 cell proliferation on plastic compared to those on the decellularised ECMs (Figure 3B). The same trend is observed in WHCO1 cell proliferation in the presence of 5-fluorouracil and epirubicin (Figure 3C,D). The presence of the decellularised ECMs appears to protect WHCO1 cells and reduce the effect of drugs on WHCO1 cancer cell growth. The effect of drugs and ECMs on WHCO1 cancer cell doubling time is shown in Table 4. For the no-drug experiment, the doubling times are similar for both plastic and ECMs, whilst the doubling times for drug and ECMs are lower than those for plastic and drugs (Table 4). Immunoblot analysis substantiated these results with Ki67 and PCNA protein levels mostly upregulated in the presence of decellularised ECMs and drugs compared to plastic dishes and drugs (Figure 3A–D; Supplemental Table S1). Thus the ECMs reduce the effect of the chemotherapeutic drugs on WHCO1 cancer cell proliferation.

### 2.4. Decellularised ECMs Reduce Drug-Induced Cell Cycle Arrest and Apoptosis in WHCO1 Cancer Cells

**Figure 4:**
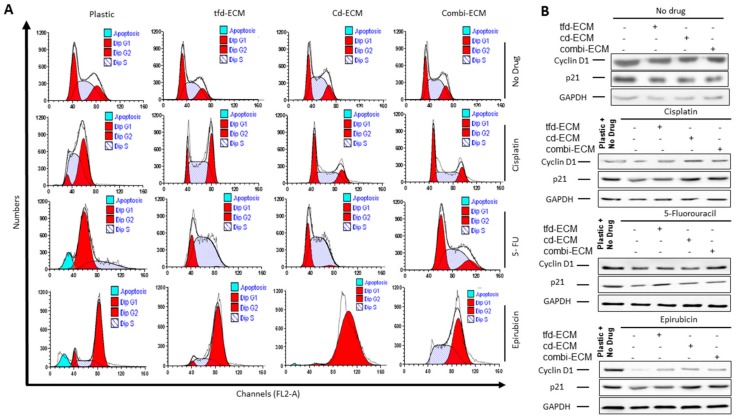
Decellularised ECMs abrogate drug-induced cell cycle arrest in WHCO1 cancer cells. (**A**) Effect of decellularised ECMs on WHCO1 cancer cell cycle progression in the presence of cisplatin, 5-fluorouracil and epirubicin. (**B**) Effect of decellularised ECMs on Cyclin D1 and p21 protein levels in WHCO1 cancer cells in response to the presence of cisplatin, 5-fluorouracil and epirubicin.

An assessment of the influence of decellularised ECMs on the effect of chemotherapeutic drugs on WHCO1 cell cycle progression and apoptosis by flow cytometry was done. With no drug present, our data indicate that the cell cycle profiles between cells on the ECMs compared to cells on plastic are the same (Figure 4A; Supplemental Table S2). Addition of cisplatin caused a G2 phase cell cycle arrest in WHCO1 cells on plastic which was abrogated by the culture of cells on decellularised ECM, with cd-ECM and combi-ECM reducing drug effect more than the tfd-ECM (Figure 4A; Supplemental Table S2). Addition of 5-flurouracil resulted in G1 phase cell cycle arrest and this effect is reduced by the presence of decellularised ECMs with WHCO1 cells cultured on combi-ECM having similar profiles as cells cultured on plastic with no drug present (Figure 4A; Supplemental Table S2). Epirubicin induced a G2 phase cell cycle arrest in WHCO1 cells on plastic and this is slightly abrogated by the presence of combi-ECM (Figure 4A; Supplemental Table S2). Immunoblot analysis of cell cycle-associated proteins show increased cyclin D1 protein levels in the presence of drugs in WHCO1 cells cultured on ECMs compared to those on plastic (Figure 4B; Supplemental Table S3).

**Figure 5:**
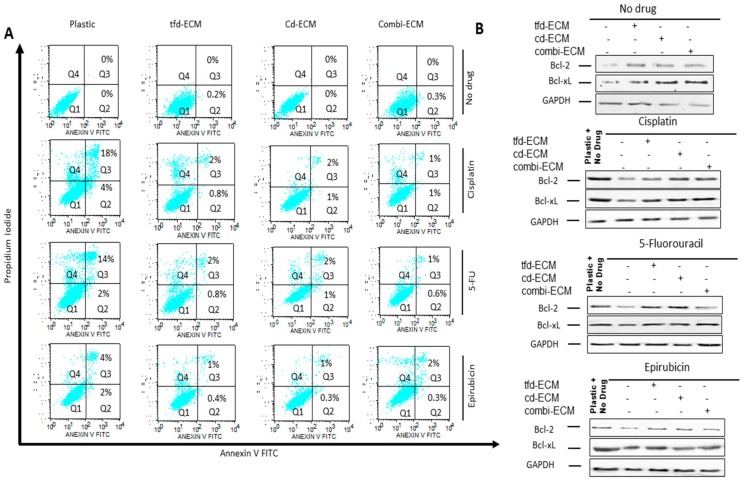
Decellularised ECMs reduce drug-induced apoptosis in WHCO1 cancer cells. (**A**) Effect of decellularised ECMs on drug-induced apoptosis in WHCO1 cancer cells (% apoptotic cells shown in quadrant Q2 and Q3). (**B**) Effect of decellularised ECMs on Bcl-2 and Bcl-xL protein levels in WHCO1 cancer cells in response to the presence of cisplatin, 5-fluorouracil and epirubicin.

**Figure 6:**
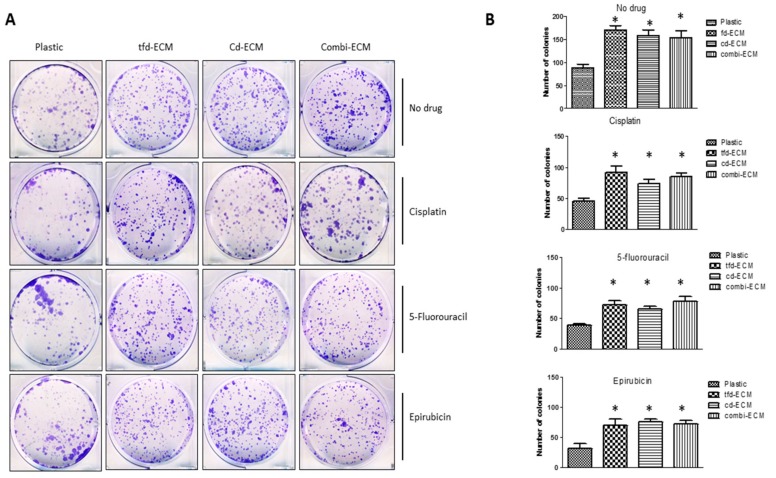
Decellularised ECMs reduce the effect of drugs on WHCO1 cancer cell colony formation. (**A**) Representative images of colony formation assay that was performed using WHCO1 cells cultured on either plastic or ECMs in the presence of cisplatin, 5-fluorouracil or epirubicin. (**B**) Quantification of colonies formed when WHCO1 cells were cultured either on plastic or ECMs in the presence of cisplatin, 5-flurouracil or epirubicin. * *p* < 0.05.

To determine whether the observed protective effect of the decellularised ECMs on WHCO1 cells was due to inhibition of apoptosis, cellular apoptosis was evaluated by Annexin V/Propidium Iodide double staining followed by flow cytometry. Culture of WHCO1 cells on decellularised ECMs reduced the number of apoptotic cells in the presence of drugs compared to cells grown on plastic (Figure 5A, shown in Q2 + Q3). Immunoblot analysis of anti-apoptotic proteins such as Bcl-2 and Bcl-xL showed an upregulation of these proteins in the presence of decellularised ECMs (Figure 5B; Supplemental Table S4). Culture of WHCO1 cells on decellularised ECMs in the presence of drugs resulted in more colonies being formed than those cultured on plastic in the presence of drugs (Figure 6A,B). A key subpopulation of tumor cells that has been found to play important roles in chemoresistance is the cancer stem cell population. We isolated cancer stem cell-like cells from cancer cells via the side population technique (Supplemental Figure S4A). We found that isolated CSC-like cells formed more colonies on ECMs and when challenged with drugs than normal cancer cells. (Supplemental Figure S4A–D). Thus, decellularised ECMs appear pro-tumorigenic.

### 2.5. Decellularised ECMs Upregulates Several Survival Pathways in WHCO1 Cancer Cells

**Figure 7:**
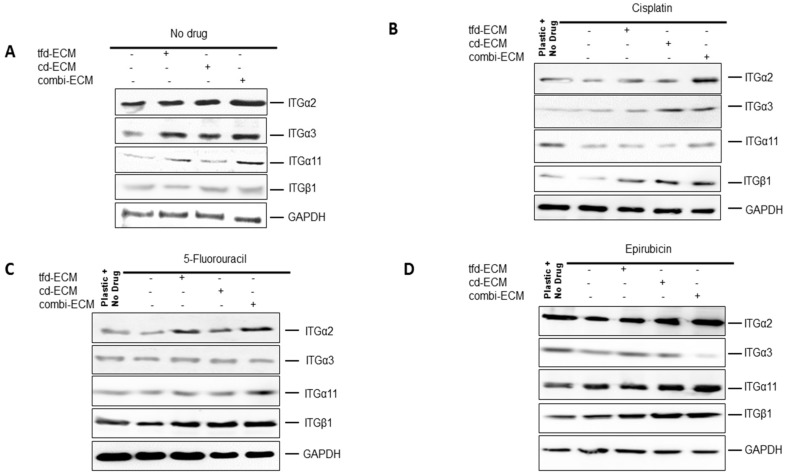
Increased integrin expression in WHCO1 cancer cells cultured on ECMs in comparison with those cultured on plastic. (**A**) Effect of decellularised ECMs on integrin α2, α3, α11 and β1 protein expression in the absence of drugs. (**B**) Effect of decellularised ECMs on integrin α2, α3, α11 and β1 protein expression in the presence of cisplatin. (**C**) Effect of decellularised ECMs on integrin α2, α3, α11 and β1 protein expression in the presence of 5-fluorouracil. (**D**) Effect of decellularised ECMs on integrin α2, α3, α11 and β1 protein expression in the presence of epirubicin GAPDH which was used as a loading control. Experiments were performed in triplicates and repeated twice.

**Figure 8:**
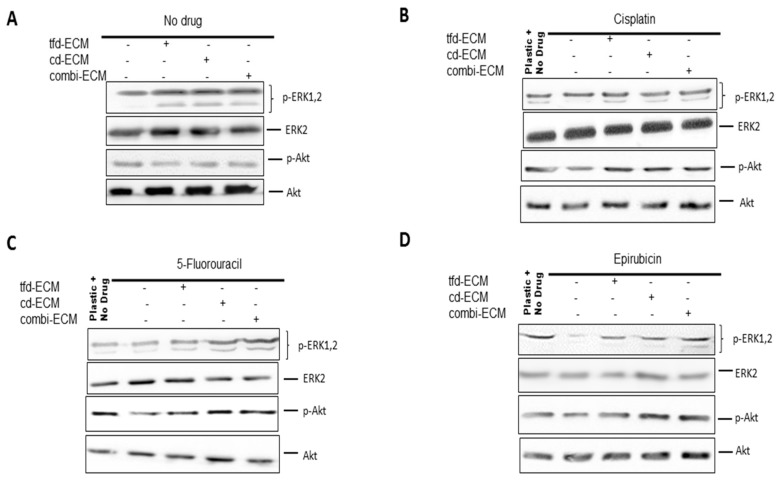
Decellularised ECMs increase both MEK-ERK and PI3K-Akt signaling activation (**A**) Influence of decellularised ECMs on MEK-ERK and PI3K-Akt signaling activation in the absence of drugs. (**B**) Influence of decellularised ECMs on MEK-ERK and PI3K-Akt signaling activation in the presence of cisplatin. (**C**) Influence of decellularised ECMs on MEK-ERK and PI3K-Akt signaling activation in the presence of 5-fluorouracil. (**D**) Influence of decellularised ECMs on MEK-ERK and PI3K-Akt signaling activation in the presence of epirubicin.

Cell surface adhesion receptors mediate most cancer cell-ECM interactions. These adhesion molecules are also responsible for transmitting extracellular initiated signaling to the cell. The levels of integrin α2, α3, α11 and β1 were assessed using immunoblot analysis. Decellularised ECMs and chemotherapeutic drugs caused differential integrin gene expression in WHCO1 cells (Figure 7A–D; Supplemental Table S5) with integrin α2 and α3 mostly upregulated compared to those on plastic and treated with drugs. These integrins are known to bind to several ECM proteins such as laminin, fibronectin, type I collagen, vitronectin and tenascin. The ECM is known to influence cellular behaviour through adhesion signaling. In addition, signal transduction pathways can be triggered by integrins resulting in the activation of several pathways affecting cancer cell proliferation, gene expression and invasion. To unravel the signaling pathways activated in cancer cells cultured on the ECMs and in response to the presence of drugs, we analysed the MEK-ERK and PI3K signaling pathways. Our data showed decellularised ECM-mediated upregulation of the MEK-ERK signaling pathway irrespective of the presence of drugs (Figure 8A–D; Supplemental Table S6). The PI3K-Akt pathway appears activated only in the presence of drugs. This is expected as PI3K-Akt signaling is one of the major survival pathways, likely activated as cancer cells respond to the presence of drugs.

### 2.6. Type I Collagen and Fibronectin Play Key Roles in WHCO1 Cancer Cell Survival and Migration In Vitro

Several studies have shown that ECM proteins are involved in the survival, migratory behaviour and the invasiveness of cancer cells. Chief among these ECM proteins are type I collagen and fibronectin. This study show that type I collagen and fibronectin are major decellularised ECM proteins and are upregulated in ESCC patient samples. Our immunohistochemical staining of ESCC samples, qRT-PCR and mass spectrometric analysis of ECMs unequivocally showed the presence of high levels of type I collagen and fibronectin. To study the role that ECM proteins play in cancer cell migration, we performed transient type I collagen and fibronectin knockdowns in transformed CT1 fibroblasts and WHCO1 cancer cells during ECM synthesis using two siRNA for each ECM protein. Both type I collagen and fibronectin knockdown through the use of siRNA showed decreased levels of both collagen and fibronectin in the media and cell lysates compared to control in transformed CT1 fibroblasts and WHCO1 cells (Supplemental Figure S5A,B).

**Figure 9:**
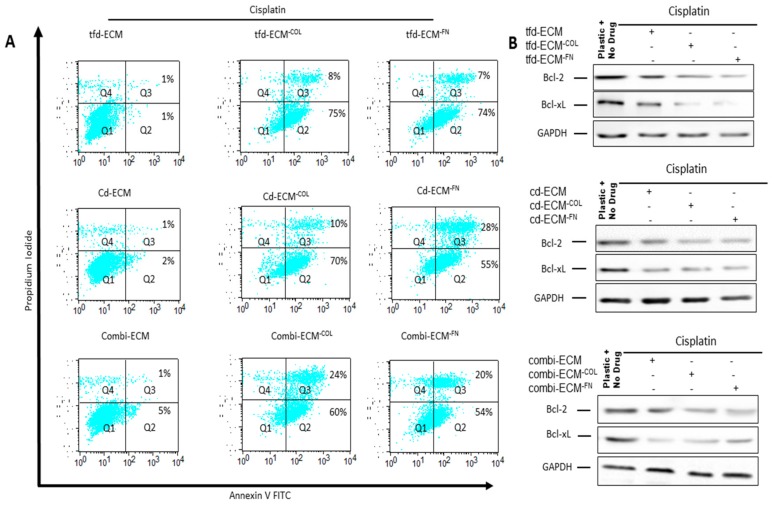
Collagen and fibronectin knockdown increase cisplatin-induced apoptosis. (**A**) Effect of collagen- and fibronectin-deficient ECMs on cisplatin-induced apoptosis in WHCO1 cancer cells (percentage of apoptotic cells shown in quadrant Q2 and Q3). (**B**) Effect of collagen- and fibronectin-deficient ECMs on Bcl-2 and Bcl-xL protein levels in the presence of cisplatin.

Type I collagen and fibronectin knockdown did not affect either fibroblasts or WHCO1 cell proliferation and morphology (data not shown). Beside the use of siRNA, the absence of ascorbic acid achieved the same knockdown of Type I collagen (data not shown). Drug-induced apoptosis is higher in cells cultured on collagen- and fibronectin-deficient ECMs than on normal decellularised ECMs (Figure 9A). Anti-apoptotic proteins such as Bcl-2 and Bcl-xL are downregulated in the absence of type I collagen and fibronectin (Figure 9B; Supplemental Table S7). Knockdown of type I collagen and fibronectin combined with challenging the cells with cisplatin resulted in less colony formation compared to cells on normal ECMs (Supplemental Figure S5C). WHCO1 cells plated on normal ECMs migrated further than those on plastic and those plated on collagen-deficient ECMs migrated slower than those on normal ECMs (Supplemental Figure S6A,B). The addition of anti-α2 blocking antibody in combination with type I collagen knockdown synergistically reduced WHCO1 cancer cell migration on combinatorial-ECM (Supplemental Figure S6C). Knockdown of fibronectin reduced migration of WHCO1 cells by around 30–50% (data not shown). This study suggests that ECM proteins such as collagen and fibronectin are mediators of cancer cell survival and migration. These results, together with the observation that WHCO1 cancer cells plated on decellularised ECMs express increased levels of both fibronectin- and type I collagen-binding integrins (ITGα2, ITGα3, ITGα5 and ITGβ1), point to the matrix as a possible therapeutic target for drugs to inhibit cancer cell growth and metastasis. Collectively our data suggest that knocking down certain ECM proteins may be effective in suppressing cancer development and enhancing chemotherapeutic effects.

## 3. Discussion

It has now been established that the tumor microenvironment plays a huge role in determining the initiation and progression of cancer [9,11,14,38,72,75,76,77,88,92,93]. The tumor microenvironment is a dynamic and ever-changing environment comprised of many components including cancer cells, fibroblasts, immune cells, endothelial cells and the ECM [38,46,47,94,95,96,97,98]. Cancer-associated fibroblasts or tumor-associated fibroblasts (CAFs or TAFs) are the major cellular component of this environment and they play a role in modulating cancer progression [99,100,101,102]. Accumulating evidence suggests that CAFs play a crucial role in tumor development and metastasis by synthesising ECM proteins. Targeting CAFs is hindered by the fact that CAFs are heterogeneous, with different subpopulations having specific phenotypes and roles during tumor development and metastasis [103,104]. Thus, targeting CAFs is challenging. Targeting the ECM proteins synthesised by resident cells within the tumor microenvironment might be an effective method to control cancer development, chemoresistance and metastasis. However, to date, few studies have included important components of the tumor microenvironment such as the ECM in their experimental setup to evaluate the interactions between cancer cells and the tumor microenvironment ECM.

In our bid to identify potential therapeutic targets within the ESCC tumor microenvironment, we evaluated how ECM components would affect the response of WHCO1 esophageal cancer cells to drugs. The first key finding of this study is that ECM proteins such as collagen and fibronectin play important roles in esophageal cancer cell survival, migration and chemoresistance. Importantly, these two ECM proteins are upregulated in esophageal tumor compared to normal tissue. The increased expression of ECM proteins including collagens, fibronectin and laminins in tumor biopsies is in contrast to the decreased expression of these ECM proteins by esophageal cancer cell lines. Thus, the increased expression of ECM proteins in tumor biopsies could be from both cancer cells and stromal cells known to be present in tumors. Several studies have shown that cancer-associated fibroblasts and macrophages synthesise increased levels of ECM proteins and these ECM proteins are associated with poor prognosis and chemoresistance in several cancer types [18,79,105,106,107,108,109,110,111,112]. Our data is in agreement with these recent studies illustrating the important role the ECM plays in the tumor microenvironment and in chemoresistance. Indeed, ECM proteins such as collagen and fibronectin have been associated with cancer cell migration before [79,113,114,115,116,117,118,119,120,121,122]. Our data show that knockdown of both collagen and fibronectin reduces esophageal cancer cell survival, migration and chemoresistance. Our data also show that MMP2 and MMP9 play a huge role in the migration of cancer cells. Several integrins were also found to be upregulated in tumor samples compared to normal samples. These ECM proteins, among other proteins, are potential chemotherapeutic targets. Several of these ECM proteins have been associated with survival pathways such as the MEK-ERK and Akt [79,123,124,125,126]. We have to appreciate that tumors are real ecosystems harbouring several cell types and non-cellular components such as the ECM. Our data suggest that the microenvironment is a shelter for cancer cells and aid their resistance to chemotherapy. Thus, the microenvironment plays a huge role in the development of chemoresistance.

Our analysis of the ECM proteins and integrins present in the stroma of the ESCC advance our understanding of the ESCC stroma and will allow future studies to focus on these proteins. The development of ESCC involves changes in the type and origin of the ECM present. Through the use of these cell-derived 3D ECMs we show that differences in the composition of different cell-derived ECMs and how this affects cancer cell response to chemotherapeutic drugs. Many features of the in vivo tumor microenvironments have been studied with many 3D tumor models having been made [127,128,129,130,131,132,133]. These models have attempted to include ECM proteins, cancer and stromal cells with the relevant biochemical and biophysical cues, into one system [2,3,90,93,134,135]. Many studies have been undertaken and have decisively shown that cells such as fibroblasts and mesenchymal stem cells are important contributors to cancer cell growth and possibly chemotherapeutic resistance [1,7,13,20,88,136,137]. Very few studies however have focused on the role the extracellular matrix play in chemotherapeutic resistance. The development of anti-stromal treatment, especially those targeting the stable ECM, which can be used together with chemotherapy, is a major advance in the treatment of several cancers.

3D ECM models have advanced our understanding of how cells interact with each other and with the ECM during tumor growth and invasion. These models have shown that cells in 3D environments show different cellular morphology and gene expression compared to those in 2D environments [9,20,88,90,93,134]. Cancer cells on 2D surfaces are normally exposed to uniform environments and concentrations of chemotherapeutic drugs whereas cells in 3D environments are exposed to gradients of biological signals and drug concentrations. Anti-cancer drugs added to cancer cells on 2D surfaces reach cancer cells without encountering physical barriers whereas cancer cells in vivo are surrounded by many tumor components and this restricts the movement of cancer drugs throughout the tumor. Earlier studies have shown that MDA MB 231 cells in 3D silk fibroin scaffolds require a higher drug dosage compared with the same cells on 2D cultures [138]. Ovarian cancer cells also show increased chemoresistance when grown as 3D spheroids compared to 2D culture [139,140,141,142]. It has now been established that 3D scaffolds generally better imitate the in vivo tumor microenvironment necessary for modelling cancer cell-ECM interactions and also for cancer cell-drug screening assays [136,143,144,145,146,147]. The interaction between cancer cells and their surrounding microenvironment plays a significant role in the acquisition of drug resistance in many cancers. The present study shows that besides physically inhibiting drugs from accessing cancer cells, the decellularised ECMs can upregulate anti-apoptotic genes such as Bcl-2 and Bcl-xL. The upregulation of these genes could be an adaptation mechanism employed by cancer cells in new environments. Thus, the decellularised ECMs influence cellular biological processes.

Our study utilised natural decellularised ECMs instead of purified ECM proteins in a bid to better mimic the in vivo tumor microenvironment [148]. In drug discovery, decellularization of cell-derived ECMs and tissues has been used as an important tool to study the interactions between the ECM and cells [18,149,150]. Done properly, decellularization can be used to successfully preserve the biochemical composition of the ECM and native tissues. By combining both cancer cells and fibroblasts we hope this will best represent the ECM milieu present in the tumor microenvironment. In addition, we also evaluated how the different ECMs affect the WHCO1 esophageal cancer cells response to commonly used drugs cisplatin, 5-fluorouracil and epirubicin. To profile early transcriptional gene expression changes, less than half of the reported IC_50_ concentrations of the drugs were used. Cisplatin is known to interfere with DNA replication, which kills mostly cancer cells as they are fast growing cells. Cisplatin-induced DNA damage activates several cellular processes culminating in the activation of cell cycle checkpoints. This results in the induction of G2/M cell cycle arrest. Our data is in agreement with literature in showing that cisplatin induces G2/M cell cycle arrest. 5-Fluorouracil is known to mediate apoptosis and induce G1/S cell cycle phase arrest. Again, our data is in agreement with this. Epirubicin acts by intercalating DNA strands and has been reported to cause both G1 and G2/M cell cycle arrest. Several reports including those using the same concentration of epirubicin as we did in this study, have shown that epirubicin induces G1 and G2/M cell cycle arrest. Our data show that epirubicin induces G2/M cell cycle arrest in WHCO1 cells. That the tumor microenvironment and the ECM are as important as the genotype of cells is becoming clearer with recent data showing the importance of the ECM in breast cancers [151,152,153,154,155,156,157]. The three ECMs used in this study were observed to be able to promote or induce resistance to chemotherapeutic drugs in esophageal cancer cell lines. It is possible that in vivo many components of the tumor microenvironment act synergistically to induce resistance to drugs and enable cancer cells to growth. That the microenvironment plays a huge role in the development of cancer might explain why certain individuals are cancer-free yet harbour oncogenic mutations. The dynamics of the relationship between cancer cells and their microenvironment will determine whether oncogenic genes and mutations will exert their function.

It has also been shown that integrin signaling driven by cell-matrix adhesion play a huge role in the development of resistance against chemotherapy-induced apoptosis and that a combination of integrin signaling inhibition and chemotherapy can lead to an improvement in cytotoxic response [158,159,160,161,162,163,164,165,166,167,168,169]. It has been shown that the interaction between ECM proteins and integrins can enhance the resistance of multiple myeloma and small cell lung cancer cells to chemotherapy [170,171]. Blocking of integrins such as β1 has been shown to sensitise breast cancer cells to treatment [172,173,174,175,176,177,178,179]. Binding of integrins to the ECM has been show to influence cell cycle progression. Several studies have shown that the binding of integrins to the ECM influence DNA repair mechanisms, with binding causing increased DNA damage repair, leading to a stable genome and cellular survival [180,181,182]. Previous studies have shown that fibroblasts-secreted type I collagen, often upregulated in tumor microenvironments, can decrease chemotherapeutic drug uptake in cancer cells thus affecting the response of the cancer cells to the drugs [183,184]. Upregulation of fibronectin was found to increase human ovarian cancer cell migration and invasion [185,186,187]. Several studies have shown that the presence of fibronectin promotes therapeutic reagents resistance in vitro [158,159,188,189]. Indeed, these studies have shown the mechanisms through which fibronectin influence carcinogenesis and chemoresistance. The source of these ECM proteins could be both tumor cells and stromal cells present within the tumor microenvironment. Small peptides that directly target the biosynthesis of ECM proteins such as fibronectin have been developed [190]. When cancer cells adhere to certain ECM proteins it has been shown they acquire chemoresistance through activation of certain survival pathways [191,192,193,194].

Our data show that the MEK-ERK and the PI3K/Akt pathways were significantly upregulated when WHCO1 cells were cultured on the different ECMs. In the presence of cisplatin, however, the MEK-ERK signaling pathway remained significantly upregulated in WHCO1 cells plated on all ECMs compared to plastic. In the presence of 5-flurouracil and epirubicin, both MEK-ERK and PI3K/Akt remained upregulated in the WHCO1 plated on the ECMs compared to plastic. Many signaling pathways such as PI3K/Akt, MEK-ERK, and the Rho/ROCK pathways have been shown to be activated when cancer cells bind to the ECM [191,195,196,197,198]. The activation of these signaling pathways could be a result of growth factors tethered on the ECMs. In breast cancer it has been shown that resistance to 5-flurouracil, epirubicin and cyclophosphamide is largely dependent on the protein composition of the stromal ECM [199]. Several cell cycle-associated proteins such as cyclin D1 are known to be induced through the activation of the MEK-ERK and PI3K-Raf signaling pathways. The activation of these Ras-mediated pathways induce the transcription of proteins such as cyclin D1 and protect it from proteolytic degradation and also export from the nucleus. Our data show the activation of the MEK-ERK and PI3K-Raf signaling pathways in the presence of ECMs. Thus, cyclin D1 might be protected from degradation by the same pathways, resulting in its presence through the G1/S/G2 cell cycle phases. The consequential effect being the protection of WHCO1 cancer cells plated on ECMs from drug-induced apoptosis as opposed to those on plastic.

Therapies that target the ECM provide a promising approach to overcome chemoresistance either by preventing ECM-conferred chemoresistance or by altering the ECM such that current therapies can overcome physical treatment limits [200,201,202,203,204]. It has been shown that the dense ECM can inhibit therapeutic drug penetration, diffusion and transport, thus the ECM acts as a barrier to drug delivery [205,206,207,208,209,210,211]. A key finding of this study is that treatments that inhibit some ECM components production such as fibronectin and type I collagen can help to achieve better drug delivery. In our study, the ECM is clearly acting as a limiting factor on drug effectiveness and we suggest combination therapy for cancer patients with one drug targeting the ECM components to aid in the diffusion of cancer drugs. Future studies should use larger patient cohorts to strengthen the results. In conclusion, we have advanced our understanding of cancer cell-ECM interaction through identifying that both type I collagen and fibronectin are involved in the proliferation and migration of esophageal cancer cells and that knocking down these two proteins can act in synergy with chemotherapeutic drugs in reducing the growth and migration of cancer cells. 

## 4. Materials and Methods

### 4.1. Clinical Tissue Collection

Twenty-one ESCC biopsy samples were collected over a period of 3 years at Groote Schuur Hospital, Cape Town, South Africa. All patients attended the oncology clinic of Groote Schuur Hospital. The biopsy samples were confirmed to be squamous cell carcinomas by a pathologist. Histological parameters were determined according to the World Health Organisation criteria. Each ESCC biopsy sample was taken together with corresponding adjacent normal tissue sample. Ethical approval was obtained from the University of Cape Town/Groote Schuur Hospital Human Research Ethics Committee (University of Cape Town, South Africa) and informed consent was obtained from all patients according to institutional guidelines. All procedures were done according to the Declaration of Helsinki guidelines. Patient biopsy samples in RNAlater solution (Qiagen, Hilden, Germany) were stored at −80 °C. RNA extraction was done as described elsewhere in the manuscript. Clinicopathological characteristics of the ESCC patients are shown in Table 1. The inclusion criteria used for tumor samples required tumor samples to contain at least 50% tumor cells.

### 4.2. Esophageal Cancer Cell Lines and Treatments

WHCO1, WHCO5 and WHCO6 cell lines were derived from biopsies of ESCC from South African patients [212]. KYSE180, KYSE450 and KYSE520 cell lines were derived from biopsies of ESCC from Japanese patients [213]. CT-1 fibroblasts are transformed fibroblasts obtained after WI38 fibroblasts are γ-radiated [91]. WI38 fibroblasts were obtained from American Type Culture Collection (USA). FG_0_, a normal skin fibroblasts were from the University of Cape Town. Cells were cultured in vitro at 37 °C in Dulbecco’s Modified Eagle’s Medium (DMEM) supplemented with 10% (*v*/*v*) fetal bovine serum (FBS), 100 U/mL penicillin and 100 µg/mL streptomycin (complete media). All cells were routinely tested for mycoplasma contamination. Preliminary data showed that the use of drug IC_50_: 5-fluorouracil (14.1 ± 3.8 µM), cisplatin (18.5 ± 6.4 µM) and epirubicin (12.8 ± 2.3 µM) would kill a considerable amount of the cells. The IC_50_ was determined as the concentration of drug needed to kill 50% of cells over 24 h. Since we were interested in investigating early transcriptional gene expression leading up to apoptosis and the response of cancer cells to chemotherapeutic drugs, we treated the cells with less than half of the reported IC_50_. WHCO1, WHCO5, WHCO6, KYSE180, KYSE 450 and KYSE 520 cells were grown overnight on plastic and on ECMs at the specified density and treated with the following concentrations of drugs: 3.5 µM 5-fluorouracil; 4.2 µM cisplatin, 2.5 µM Epirubicin and 0.1% DMSO (control) [214].

### 4.3. Preparation of Decellularised ECMs and ECM Coatings

Decellularised ECMs were prepared from transformed CT-1 fibroblasts and WHCO1 esophageal cancer cells as previously described [20,87,90,215]. All cells were cultured at 37 °C in complete media. Ascorbic acid was added at a final concentration of 50 μg/mL every alternate day. Cells were maintained up to 4 days post confluence. Decellularization was achieved by the addition of 20 mM ammonium hydroxide for 1 min. The ECMs were incubated for an hour with DNAse I (10 U/mL). The resulting ECMs were washed three times with sterile PBS. Sterilisation was achieved by exposing the dishes to UV light. Dishes coated with decellularised ECMs were used immediately or stored at 4 °C. Transformed CT1 fibroblasts produced transformed fibroblast-derived ECM (tfd-ECM), WHCO1 cancer cells produced cancer-derived ECM (cd-ECM) and co-cultured transformed CT1 fibroblasts and WHCO1 cancer cells produced combinatorial ECM (combi-ECM). Decellularised ECMs without fibronectin (ECM^-FN^) and without Type I collagen (ECM^-COL^) were produced by transfecting transformed CT-1 fibroblasts and WHCO1 cells with either fibronectin siRNA or COL1A1 siRNA during ECM synthesis. To maintain the knockdown of type I collagen and fibronectin during ECM synthesis, subsequent transfection of cells with fibronectin siRNA and COL1A1 siRNA was done after 3 days. To characterise the ECMs, 5 M Guanidine-HCL in buffer was added to the ECM solution and the resulting protein was run on a SDS-PAGE for an hour. Total protein was stained with Ponceau stain and Coomassie stain. Human fibronectin and type I collagen were also loaded to serve as markers.

### 4.4. Cell Cytotoxicity Assay

Cell growth curves or proliferation rates after plating WHCO1 on decellularised ECMs and/or treatment with cisplatin, 5-fluro-uracil and epirubicin drugs were determined using the Countess Counter (Thermo Fisher Scientific, Waltham, MA, USA). WHCO1 cells (5 × 10^5^) were plated on the decellularised ECMs and/or treated with the drugs for the indicated time periods. Cells were trypsinised and centrifuged at 1800 rpm for 5 min. Cells were suspended in 2 mL complete media and 10 µL was mixed with Trypan Blue. In addition, WHCO1 cells were plated in 96-well plates with or without the ECMs and allowed to grow for about 48–72 h in either drug-free medium or under treatment with increasing concentrations of cisplatin, 5-fluro-uracil and epirubicin drugs. The IC_50_ for each cell population was measured using the MTT assay. Briefly, WHCO1 cells were plated in 96-well plates with or without ECMs overnight. Drugs were added and incubation continued for 24 h. The MTT reagent was added and the cells were shaken. Colour changes were read on a microplate reader.

WHCO1 cancer cell doubling time (PD) is the time it takes the population of WHCO1 cells to double and was calculated based on the following formula:

PD = t × ln2/ln (FCC/SCC)

where t equals time in hours, ln represents the natural logarithm, FCC represents the final WHCO1 cancer cell number, and SCC represents the starting WHCO1 cancer cell number.

### 4.5. Quantitative Real Time RT-PCR

Fresh ESCC biopsy specimens were cut into pieces for RNA extraction using the Qiazol reagent (BioRad, Munich, Germany) and the quality of the RNA was checked by electrophoresis on a 2% agarose gel. For in vitro experiments, WHCO1 cells were treated as described above and RNA was extracted using Qiazol reagent based on the procedure of Chomczynski and Sacchi [216]. Total RNA (100–500 ng) was used to synthesise complementary DNA (cDNA) using Improm II reverse transcriptase (Promega, Madison, WI, USA). cDNA from triplicate samples were analyzed using qRT-PCR reactions and monitored using a Light Cycler 480 II machine. Thermocycling was performed under standard conditions with an initial denaturation step of 5 min at 95 °C, 30–40 cycles of denaturation, annealing and extension at 72 °C. Primers used in the study are listed in Supplemental Table S8. The 2^−ΔΔCt^ method was used to compute the relative gene expression for each sample by comparing to control cells [217]. Differences in gene expression for biopsy samples are shown as fold changes in tumor tissues (T) compared to the corresponding normal tissues (N) (given as 1, red line) from the same patient. GAPDH was used as a normaliser for both biopsy specimens and in vitro experiments. The Mann-Whitney test (2-tailed, non-parametric) was used to compare significance differences in gene expression between tumor and normal tissues. *p* value was set at *p* < 0.05 to be considered statistically significant. 

### 4.6. Immunoblot Analysis

Immunoblot analysis was done according to standard protocols. Cell lysates were obtained by lysing cells with RIPA buffer in the presence of protease inhibitor cocktail. Total protein concentration was determined using the BCA kit and BSA as a standard. Cell lysates (50 μg) were separated by electrophoresis on a 10% polyacrylamide/SDS gels under reducing conditions (50 mM β-mercaptoethanol). Proteins were transferred to a nitrocellulose membranes for 1 h at 4 °C. Membranes were blocked with 5% fat-free milk in Tris Buffered Saline (TBS) containing Tween-20. The membranes were incubated overnight at 4 °C with the following primary antibodies: anti-Ki67 antibody, anti-PCNA antibody, anti-Cyclin D1 antibody, anti-*p*-ERK1/2 (Thr202/Tyr204), anti-ERK2, anti-Akt, anti-*p*-Akt, anti-p21, anti-p53, anti-Bcl-2, anti-Bcl-xL antibody, anti-MMP-2 antibody, anti-MMP-9 antibody, anti-ITGα2 antibody, anti-type I collagen antibody, anti-fibronectin antibody, anti- ITGα3 antibody, anti- ITGα11 antibody, anti-ITGβ1 and anti-GAPDH antibody. Three washes were done using TBS-Tween buffer and then the membranes were incubated with secondary antibodies conjugated to horse radish peroxidase (HRP). Detection was done using Lumiglo substrate (KPL, Gaithersburg, MD, USA). All experiments were done in triplicates and repeated at least twice.

### 4.7. Cell Cycle and Colony Formation Assay

WHCO1 cancer cells (5 × 10^5^) were cultured on plastic and on decellularised ECMs and treated with drugs for the indicated incubation times. Cells were then dissociated from culture plates using trypsin-EDTA and processed for flow cytometry analysis. Cells were washed twice with cold PBS and fixed with 70% ethanol for 30 min at 4 °C. Washing was done twice with PBS and RNase A (10 µg/mL) was also added for 3 h at 4 °C. Cells were stained with propidium iodide solution (1 mg/mL) and analyzed with a FACScan cell sorter (BD Biosciences, Franklin Lakes, NJ, USA). Ten-thousand cells were collected and the cell cycle profiles were calculated using the Cellquest Software. For colony formation, WHCO1 cells were plated on plastic and on decellularised ECMs in 6-well plates at 500 cells per well and incubated for 10 days. Methanol (100%) was used to fix the cells and cells were stained with 0.5% crystal violet. Colonies were counted using UVP software (Upland, CA, USA) and the numbers were plotted on a graph. Images were taken using a camera. The experiments were performed at least three times.

### 4.8. Annexin V/Propidium Iodide Assay for Apoptosis

WHCO1 cell apoptosis was evaluated using double staining with Annexin V conjugated to Fluorescein isothiocyanate (FITC) and Propidium Iodide (PI). After incubation, cells were washed in PBS from each treatment setup and the Annexin binding buffer was used for re-suspension. Cells were stained with Annexin V conjugated to FITC and PI following the manufacturer’s instructions. Annexin binding buffer was used to wash cells and resuspension was done in 4% paraformaldehyde (PFA). Cells were incubated for 15 min at 25 °C in the dark. Flow cytometric analysis was done using a Beckman Coulter Flow Cytometer (Beckman Coulter, Life Sciences, Indianapolis, IN, USA). Data acquisition (2 × 10^4^ events per treatment condition) was performed using the Cellquest software (Version 5.1, Becton Dickinson, Franklin Lakes, NJ, USA).

### 4.9. siRNA Transfection Assay

Short interfering RNAs (siRNAs) were purchased from Santa Cruz Biotechnology (Santa Cruz, CA, USA). siRNAs were dissolved in transfection diluent according to the manufacturer’s protocols. Transformed CT1 fibroblasts and WHCO1 cancer cells were transfected with COL1A1 and Fibronectin siRNAs using Transfectin reagent (BioRad, Hercules, CA, USA) and ECM synthesis continued for the indicated period. To maintain knockdown efficiency, subsequent transfections were carried out every 3 days for the duration of the ECM synthesis. In a separate experiment, type I collagen knockdown was achieved by removing ascorbic acid during ECM synthesis with a similar result compared to the use of COL1A1 siRNA. Cells were cultured in 6-well plates with or without the addition of ascorbic acid and ECM synthesis was continued for the duration indicated above [218]. Confirmation of type I collagen and fibronectin knockdowns was done using immunoblot and SDS PAGE analysis.

### 4.10. Immunofluorescence

ECMs were synthesised on glass coverslips as described before. Cells were then plated on the ECM for the duration indicated elsewhere. Fixing of the cells was achieved using 3% paraformaldehyde solution. Permeabilisation of the cells was done using 0.1% Triton X-100 (Sigma Aldrich Chemie, Steinheim, Germany) in PBS. Blocking of cells was done using 1% BSA for 1 h at room temperature and then incubated with various primary antibodies overnight at 4 °C. Cells were then washed three times with PBS. Secondary antibodies were conjugated to FITC and Fluor 488. DAPI was added in order to visualise the nucleus. Fluorescence was observed using a Zeiss Inverted Microscope with a 20× objective. Acquisition of images was achieved using the CellSens Imaging System (Olympus, Tokyo, Japan). Proteoglycan composition within the ECMs was detected by staining the ECMs with 1% Alcian blue after preparation using a standard protocol. Briefly ECM synthesis and preparation was done on coverslips. ECMs were incubated with 1% Alcian Blue solution for 20 min and washed three times. Images were observed and photographed using a light microscope (Olympus CKX41 with SC30 camera). Images were taken at 100× magnification. 

### 4.11. Migration Assay

Confluent cells were trypsinised and neutralised by adding DMEM supplemented with 10% FCS and Penicillin and streptomycin. Centrifugation was done at 1800 rpm for 5 min at 25 °C and cells were resuspended in DMEM. The Countess Cell Counter was used for cell counting to give a final cell density of 5000 cells per microliter. Cellular foci of 4 µL containing a total of 20,000 cells were added to plastic dishes or to the ECMs. To prevent cell death due to evaporation of media, an extra 100 µL of DMEM media was added to the cellular foci and incubation continued for 2 h. A further 3 mL of DMEM with 10% FCS was then added and incubation continued for 24 h. In a separate experiment, cells were incubated with anti-α2 integrin blocking antibody for 30 min before plating. Images were taken at 0 h and at 24 h with a microscope. Image J (version 1.52g, National Institutes of Health; Bethesda, MD, USA) was then used to measure the area of migrating mass of cells. Migration on ECMs was normalised to that on plastic dishes.

### 4.12. Immunohistochemistry

Formalin-fixed, paraffin-embedded whole sections of esophageal tumor and normal samples were used to quantify type I collagen. Sections were of 4 µm thickness and standard histological analysis was carried out. Paraffin slides were deparaffinised in xylene and rehydration was achieved through the use of graded alcohols. Slides were heated in 0.01 M citrate buffer (pH 6.0) in a bath for 20 min at 97 °C for antigen retrieval. Slides were allowed to cool and were rinsed in TBS; the endogenous peroxidase was inactivated with 3% hydrogen peroxide. After protein block, incubation of slides with a primary antibody against human type I collagen was done for 1 h. TBS was used to rinse sections and incubation was done for 20 min with biotinylated secondary antibodies. Sections were rinsed with TBS and incubated with streptavidin-HRP. Peroxidase reactivity was visualised using a 3,3-diaminobenzidine (DAB). Counterstaining of sections was done with haematoxylin. Sections were mounted and images were obtained using a light microscope. Whole tissue sections from tumor and normal blocks were stained and compared by visual inspection. Results were evaluated independently by two observers.

### 4.13. Mass Spectrometry

The proteomic profiles of the 3D ECMs were assessed using mass spectrometry. Mass spectrometry analysis was performed on duplicate ECM samples. The samples were prepared by filter aided sample preparation (FASP) according to Wisniewski et al. [219]. Reduced and alkylated protein samples were tryptically digested at a 1:50 enzyme to sample ratio overnight for 16 h in a wet chamber at 37 °C. For each sample, 10 µg aliquots of the resulting tryptic peptides were acidified in 0.1% formic acid and desalted using in-house produced C18 stage tips. The samples were dried in a vacuum concentrator, and reconstituted in 2% acetonitrile with 0.1% formic acid prior to LC-MS/MS analysis. Samples were analysed on a Thermo Scientific Dionex Ultimate 3000 UHPLC (Thermo Fisher Scientific, Waltham, MA, USA) coupled to a Thermo Scientific Q Exactive hybrid quadrupole Orbitrap mass spectrometer. The samples were loaded onto a 2 cm Luna C18 100 µM internal diameter fused silica pre-column, packed in-house, and then separated on a 40 cm Aeris peptide C18 75 µM internal diameter analytical column, packed in-house. A total of 400 ng of each sample was analysed on a 70 min gradient from 6–40% acetonitrile with a flow rate of 400 nL/min at 40 °C. The nanoelectrospray voltage was set to 2.2 kV, and the capillary temperature was 320 °C. The Q Exactive was operated in data-dependent mode, with full scan MS spectra (*m*/*z* 300–1750) acquired in the Orbitrap analyser after accumulation to an AGC target of 3e6 or an injection time of 250 ms at a resolution of 70,000. The 10 most intense peptide ions were sequentially isolated and fragmented by HCD and acquired at a resolution of 17,500. Dynamic exclusion was enabled after 30s and for a repeat count of one. The raw files were processed using MaxQuant version 1.2.7.429 (Computational Systems Biochemistry, Martinsried, Germany) and the MS/MS spectra were searched using the Andromeda search engine against the Uniprot human protein database. The initial maximal allowed mass tolerance was set to 20 ppm for the first search and then to 4.5 ppm in the main search, and 20 ppm for fragment ions. Enzyme specificity was set to trypsin with a maximum of two missed cleavages. Carbamidomethylation of cysteine was set as a fixed modification, and oxidation of methionine and protein N-terminal acetylation were selected as variable modifications. The minimum peptide length was set to seven amino acids. Label-free protein quantification was performed using the label-free quantification (LFQ) algorithm implemented in the MaxQuant software (version 1.1.1.25, Computational Systems Biochemistry, Martinsried, Germany) with a 2 min window for matching between runs and, a maximum 1% peptide and 1% protein false discovery rate. Protein intensity values were normalized automatically using the LFQ algorithm to identify differentially expressed proteins. Bioinformation analyses of the data were performed using Perseus v.1.2.7.4 software. Reverse and “only identified by site” entries were excluded. LFQ intensity values were log2 transformed, and the dataset was filtered to contain only entries with two minimum valid values in at least one group. Statistical significance was assessed using Student’s *t*-test to identify differentially expressed proteins between the groups.

### 4.14. Statistical Analysis

Statistical analysis was performed using GraphPad Prism software. The Mann-Whitney test (2-tailed, non-parametric) was used to compare significance differences in gene expression between tumor and normal tissues. *p* value was set at *p* < 0.05 to be considered statistically significant. Evaluation of statistical significance between control cells and cells plated on plastic or ECM/treated with chemotherapeutic drugs was done using the paired Student’s *t* test. *p* value was set at *p <* 0.05 to be considered statistically significant. Correlation coefficients were determined using Pearson’s correlation coefficient in Microsoft Excel. Pearson’s correlation coefficients were calculated to determine a point estimate of the strength of the association between the different ECM preparations.

## Figures and Tables

**Table 1:** Clinicopathological characteristics of 21 ESCC samples from patients used in the study.

Biopsy Number	Histology	Sex	Age	Tumor Differentiation (Grade)	Tumor Site ICD-10	Invasive or Infiltrating
543	ESCC	M	55	ND	C15.4	Infiltrating
547	ESCC	F	30	Moderate	C15.5	Invasive
551	ESCC	M	47	Moderate	C15.5	Invasive
556	ESCC	F	54	Moderate	C15.4	Invasive
561	ESCC	M	58	Moderate	C15.9	Keratinizing
563	ESCC	M	52	Moderate	C15.5	Infiltrating
569	ESCC	F	79	Poor	C15.4	Invasive
571	ESCC	F	48	Moderate	C15.3	Keratinizing
573	ESCC	F	41	ND	C15.3	Infiltrating
591	ESCC	M	47	Moderate	C15.4	Invasive
596	ESCC	F	67	Moderate	C15.4	Invasive
601	ESCC	M	59	ND	C15.4	Infiltrating
607	ESCC	F	48	Moderate	C15.4	ND
613	ESCC	M	54	Moderate	C15.9	Invasive
618	ESCC	F	60	Moderate	C15.4	Keratinizing
619	ESCC	M	57	Moderate	C15.4	Infiltrating
621	ESCC	F	64	Moderate	C15.4	Invasive
622	ESCC	F	83	ND	C15.4	Infiltrating
627	ESCC	M	52	Moderate	ND	ND
634	ESCC	F	57	Moderate	C15.4	Keratinizing
635	ESCC	M	57	Moderate	C15.4	Keratinizing

ESCC: Esophageal squamous cell carcinoma; M: Male; F: Female; ND: Not done.

**Table 2:** Major ECM proteins and associated entities identified in decellularised ECMs by mass spectrometric analysis.

Glycoproteins	Collagens	ECM Regulators	ECM Affiliated Proteins	Secreted Factors	Proteoglycans
Gene Name					
*FN1*	*COL1A1*	*TGM2*	*LGALS1*	*S100A13*	*HSPG2*
*LAMA3*	*COL1A2*	*HTRA1*	*FREM2*	*EGFL7*	*BGN*
*LAMA5*	*COL6A3*	*CSTB*	*ANXA2*	*IGF2*	*DCN*
*FBN1*	*COL3A1*	*LOXL2*	*FREM1*	*S100A11*	*LUM*
*TGFB1*	*COL12A1*	*LOXL1*	*ANXA6*	*S100A6*	*ASPN*
*TNC*	*COL6A1*	*SERPINH1*	*ANXA5*	*S100A13*	*OGN*
*EMILIN1*	*COL4A2*	*CTSB*	*COLC12*	*CXCL12*	*PRELP*
*LAMC1*	*COL6A2*	*LOX*	*CLEC3B*	*CCL25*	*VCAN*
*LAMB2*	*COL4A5*	*ITH5*	*LGALS3*	*PF4*	
*FBLN2*	*COL4A4*	*ADAM10*	*LGALS8*	*FGF2*	
*LAMA2*	*COL5A2*	*ADAMTSL1*	*SEMA3C*	*INSL5*	
*TNXB*	*COL7A1*	*PLG*	*CLEC14A*	*ANGPTL2*	
*POSTN*	*COL11A1*	*PZP*	*ANXA9*	*S100A9*	
*THBS1*	*COL4A1*	*CTSK*	*ANXA1*		
*FBN2*	*COL5A1*	*ADAMTSL5*	*PLXDC2*		
*FBLN1*	*COL5A3*	*SERPINA1A*	*SFTPA1*		
*LAMB3*	*COL14A1*	*SERPINA3K*	*CSPG4*		
*LAMA4*	*COL16A1*	*PLOD1*	*SFTPD*		
*AGRN*	*COL18A1*				
*FGB*	*COL15A1*				
*LAMC2*					
*VWF*					
*HMCN1*					
*LTBP4*					

**Table 3:** Cytotoxicity quantification. Oesophageal cancer cells, WHCO1, were treated with drugs as indicated and the effect was evaluated by the MTT assay. The IC_50_ was determined as the concentration of drug needed to kill 50% of cells over 24 h treatment. Values of the IC_50_ are shown as mean ± S.D. of three independent determinations.

Drug	Plastic	tfd-ECM	cd-ECM	combi-ECM
Cisplatin (IC_50_ ± S.D. (µM))	18.5 ± 6.4	23.8 ± 3.2	22.4 ± 4.5	25.7 ± 3.2
5-FU (IC_50_ ± S.D. (µM))	14.1 ± 3.8	19.1 ± 2.6	20.6 ± 2.2	21.9 ± 1.8
Epirubicin (IC_50_ ± S.D. (µM))	12.8 ± 2.3	17.3 ± 4.5	18.5 ± 1.9	27.8 ± 5.3

**Table 4:** Average esophageal cancer cells, WHCO1, population doubling times were calculated as described in Materials and Methods. Doubling times are presented as mean ± S.D of three independent determinations.

	Plastic	tfd-ECM	cd-ECM	combi-ECM
No Drug (hours)	33.6 ± 3.3	38.6 ± 5.7	37.1 ± 4.2	36.8 ± 4.5
Cisplatin (hours)	55.3 ± 9.4	39.5 ± 4.3	36.9 ± 3.8	36.7 ± 5.8
5-FU (hours)	56.2 ± 5.1	39.5 ± 3.6	32.6 ± 4.6	31.9 ± 3.8
Epirubicin (hours)	58.3 ± 2.5	34.7 ± 3.5	30.7 ± 4.9	32.1 ± 3.8

## Supplementary Materials

Supplementary Materials can be found at http://www.mdpi.com/1422-0067/19/10/2861/s1.

## Abbreviations

3D	Three dimensional
BSA	Bovine serum albumin
DNA	Deoxyribonucleic acid
DMSO	Dimethyl sulfoxide
EDTA	Ethylenediaminetetraacetate
ECM	Extracellular matrix
ESCC	Esophageal squamous cell carcinoma
FN	Fibronectin
5-FU	5-fluorouracil
ITG	Integrin
Tfd-ECM	Transformed fibroblast-derived ECM
cd-ECM	Cancer cell-derived ECM
combi-ECM	Combinatorial-ECM
MS	Mass spectrometry.
MMP	Matrix metalloprotease
PAGE	Polyacrylamide gel electrophoresis
SDS	Sodium dodecyl sulphate
