# Supplementary material for: The Role of Tumor Microenvironment in Chemoresistance: 3D Extracellular Matrices as Accomplices
Source: Int J Mol Sci. 2018 Sep 20;19(10):2861. doi: 10.3390/ijms19102861 (PMC6213202; doi:10.3390/ijms19102861)
Supplement: Supplementary file 1 [file ijms-19-02861-s001.pdf]

Supplemental Table S1. Relative densitometric quantification of immunoblot band intensities of at least 2 immunoblot gels as illustrated in Figure 3 for the ratio of protein normalized to GAPDH levels. Statistical significance refers relative to either Plastic or Plastic and No drug which is taken as 1. Data is shown as mean ± S.D.

		No drug (A)			
		Plastic	Tfd-ECM	cd-ECM	Combi-ECM
Ki67		1	1.1 ± 0.1	1.0 ± 0.1	1.1 ±0.1
PCNA		1	1.2 ± 0.2	1.0 ± 0.1	0.9 ± 0.2
		Cisplatin (B)			
	Plastic + No drug	Plastic	Tfd-ECM	cd-ECM	Combi-ECM
Ki67	1	0.4 ± 0.1*	0.8 ± 0.2	0.6 ± 0.1*	0.8 ± 0.2
PCNA	1	0.3 ± 0.1*	0.8 ± 0.2	0.7 ± 0.2*	0.8 ± 0.2
		5-Fluorouracil (C)			
	Plastic + No drug	Plastic	Tfd-ECM	cd-ECM	Combi-ECM
Ki67	1	0.3 ± 0.1*	0.5 ± 0.2*	0.4 ± 0.1*	0.8 ± 0.2
PCNA	1	0.3 ±0.1 *	0.4 ± 0.2*	0.4 ± 0.1*	0.6 ± 0.3*
		Epirubicin (D)			
	Plastic + No drug	Plastic	Tfd-ECM	cd-ECM	Combi-ECM
Ki67	1	0.5 ± 0.1*	1.1 ± 0.2	0.8 ± 0.2	0.9 ± 0.1
PCNA	1	0.3 ± 0.1*	0.6 ± 0.2*	0.8 ± 0.2	0.8 ± 0.2

* $p < 0.05$.

Supplemental Table S2. Cell cycle analysis data showing percentage of cells in G1 phase, G2 phase and S phase and those undergoing apoptosis as shown in Figure 4A. Values are shown as single data of one of two determinations done.

		Plastic	Tfd-ECM	cd-ECM	Combi-ECM
No drug	G1 phase (%)	32.8	35.5	30.5	30.4
	G2 phase (%)	21.9	17.8	16.7	16.9
	S phase (%)	45.3	46.7	52.8	52.7
	Apoptosis (%)	0	0	0	0
Cisplatin	G1 phase (%)	5.3	11.3	34.7	33.3
	G2 phase (%)	45.0	31.7	18.5	18.4
	S phase (%)	49.1	57.0	46.8	48.3
	Apoptosis (%)	0.6	0	0	0
5-fluorouracil	G1 phase (%)	73.0	22.0	26.3	37.7
	G2 phase (%)	0.4	1.4	2.1	13.4
	S phase (%)	26.7	76.6	71.6	48.9
	Apoptosis (%)	13.9	0	0	0
Epirubicin	G1 phase (%)	8.4	3.3	1.4	0
	G2 phase (%)	71.2	74.9	98.6	52.6
	S phase (%)	20.4	21.8	0	47.4
	Apoptosis (%)	13.3	1.0	0.5	0

Supplemental Table S3. Relative densitometric quantification of immunoblot band intensities of at least 2 immunoblot gels as illustrated in Figure 4B for the ratio of protein normalized to GAPDH levels. Statistical significance refers relative to either Plastic or Plastic and No drug which is taken as 1. Data is shown as mean ± S.D.

		No drug (top panel)			
		Plastic	Tfd-ECM	cd-ECM	Combi-ECM
Cyclin D1		1	1.1 ± 0.2	1.0 ± 0.2	1.1 ± 0.1
p21		1	0.5 ± 0.1*	0.4 ± 0.2*	0.5 ± 0.2*
		Cisplatin			
	Plastic + No drug	Plastic	Tfd-ECM	cd-ECM	Combi-ECM
Cyclin D1	1	0.4 ± 0.1*	0.8 ±0.2	1.1 ± 0.1	1.0 ± 0.2
p21	1	0.4 ± 0.2*	0.8 ± 0.2	1.0 ± 0.1	0.9 ± 0.2
		5-Fluorouracil			
	Plastic + No drug	Plastic	Tfd-ECM	cd-ECM	Combi-ECM
Cyclin D1	1	0.3 ± 0.1*	0.5 ± 0.1*	0.4 ± 0.1*	0.8 ± 0.2
p21	1	0.4 ± 0.1*	0.6 ± 0.2*	0.6 ± 0.1*	0.7 ± 0.2*
		Epirubicin (lower panel)			
	Plastic + No drug	Plastic	Tfd-ECM	cd-ECM	Combi-ECM
Cyclin D1	1	0.2 ± 0.1*	0.5 ± 0.2*	0.4 ± 0.2*	0.6 ± 0.2*
p21	1	0.5 ± 0.1*	0.8 ± 0.2	1.0 ± 0.2	1.1 ± 0.2

* $p < 0.05$.

Supplemental Table S4. Relative densitometric quantification of immunoblot band intensities of at least 2 immunoblot gels as illustrated in Figure 5B for the ratio of protein normalized to GAPDH levels. Statistical significance refers relative to either Plastic or Plastic and No drug which is taken as 1. Data is shown as mean ± S.D.

		No drug (top panel)			
		Plastic	Tfd-ECM	cd-ECM	Combi-ECM
Bcl-2		1	1.5 ± 0.2*	1.4 ± 0.1*	1.3 ± 0.2*
Bcl-xL		1	1.4 ± 0.1*	1.7 ± 0.2*	1.7 ± 0.1*
		Cisplatin			
	Plastic + No drug	Plastic	Tfd-ECM	cd-ECM	Combi-ECM
Bcl-2	1	0.2 ± 0.0*	0.4 ± 0.1*	1.0 ± 0.2	0.9 ± 0.2
Bcl-xL	1	0.3 ± 0.0*	0.8 ± 0.2	0.7 ± 0.1*	0.8 ± 0.2
		5-Fluorouracil			
	Plastic + No drug	Plastic	Tfd-ECM	cd-ECM	Combi-ECM
Bcl-2	1	0.1 ± 0.0*	1.1 ± 0.0	1.0 ± 0.1	0.5 ± 0.2*
Bcl-xL	1	0.4 ± 0.0*	0.9 ± 0.1*	0.8 ± 0.1*	1.0 ± 0.1
		Epirubicin (lower panel)			
	Plastic + No drug	Plastic	Tfd-ECM	cd-ECM	Combi-ECM
Bcl-2	1	0.6 ± 0.1	1.0 ± 0.1	0.8 ± 0.2	0.7 ± 0.1*
Bcl-xL	1	0.3 ± 0.0*	0.6 ± 0.2*	0.8 ± 0.2	0.8 ± 0.2

* $p < 0.05$.

Supplemental Table S5. Relative densitometric quantification of immunoblot band intensities of at least 2 immunoblot gels as illustrated in Figure 7 for the ratio of protein normalized to GAPDH levels. Statistical significance refers relative to either Plastic or Plastic and No drug which is taken as 1. Data is shown as mean ± S.D.

		No drug (A)			
		Plastic	Tfd-ECM	cd-ECM	Combi-ECM
ITGα2		1	1.0 ± 0.1	1.2 ± 0.1*	1.3 ± 0.1*
ITGα3		1	1.4 ± 0.1*	1.3 ± 0.1*	1.5 ± 0.1*
ITGα11		1	1.3 ± 0.2*	1.0 ± 0.1	1.4 ± 0.2*
ITGβ1		1	1.0 ± 0.1	1.2 ± 0.2	1.1 ± 0.1
		Cisplatin (B)			
	Plastic + No drug	Plastic	Tfd-ECM	cd-ECM	Combi-ECM
ITGα2	1	0.2 ± 0.0*	1.1 ± 0.1*	1.0 ± 0.2	1.4 ± 0.2*
ITGα3	1	1.0 ± 0.0*	1.2 ± 0.1*	1.4 ± 0.0*	1.5 ± 0.2*
ITGα11	1	0.2 ± 0.0*	0.1 ± 0.1*	0.1 ± 0.0*	0.4 ± 0.1*
ITGβ1	1	1.1 ± 0.0*	1.3 ± 0.0*	1.4 ± 0.2*	1.5 ± 0.1*
		5-Fluorouracil (C)			
	Plastic + No drug	Plastic	Tfd-ECM	cd-ECM	Combi-ECM
ITGα2	1	0.3 ± 0.0*	1.3 ± 0.1*	1.2 ± 0.1*	1.5 ± 0.2*
ITGα3	1	0.8 ± 0.1*	0.9 ± 0.1	1.1 ± 0.0*	0.7 ± 0.1*
ITGα11	1	0.3 ± 0.0*	1.2 ± 0.0*	1.0 ± 0.0	1.6 ± 0.1*
ITGβ1	1	0.4 ± 0.0*	1.0 ± 0.1	1.2 ± 0.1*	1.4 ± 0.1*
		Epirubicin (D)			
	Plastic + No drug	Plastic	Tfd-ECM	cd-ECM	Combi-ECM
ITGα2	1	0.2 ± 0.0*	0.4 ± 0.1*	1.0 ± 0.1	1.3 ± 0.2*
ITGα3	1	0.4 ± 0.0*	0.8 ± 0.1*	0.4 ± 0.1*	0.2 ± 0.0*
ITGα11	1	1.0 ± 0.1	1.0 ± 0.1	1.0 ± 0.1	1.3 ± 0.2*
ITGβ1	1	1.0 ± 0.1	1.2 ± 0.1*	1.3 ± 0.1*	1.4 ± 0.2*

* $p < 0.05$.

Supplemental Table S6. Relative densitometric quantification of immunoblot band intensities of at least 2 immunoblot gels as illustrated in Figure 8 for the ratio of protein normalized to ERK2 and Akt2 levels. Statistical significance refers relative to either Plastic or Plastic and No drug which is taken as 1. Data is shown as mean ± S.D.

		No drug (A)			
		Plastic	Tfd-ECM	cd-ECM	Combi-ECM
p-ERK 1,2		1	1.2 ± 0.1*	1.2 ± 0.0*	1.3 ± 0.1*
p-Akt		1	1.0 ± 0.1	1.0 ± 0.2	1.0 ± 0.1
		Cisplatin (B)			
	Plastic + No drug	Plastic	Tfd-ECM	cd-ECM	Combi-ECM
p-ERK 1,2	1	0.5 ± 0.0*	1.2 ± 0.1*	1.2 ± 0.1*	1.5 ± 0.1*
p-Akt	1	0.4 ± 0.0*	1.2 ± 0.0*	1.3 ± 0.2*	1.4 ± 0.0*

		5-Fluorouracil (C)			
	Plastic + No drug	Plastic	Tfd-ECM	cd-ECM	Combi-ECM
p-ERK 1,2	1	1.0 ± 0.0	1.2 ± 0.0*	1.4 ± 0.2*	1.6 ± 0.2*
p-Akt	1	0.3 ± 0.0*	0.9 ± 0.1	1.0 ± 0.0	1.2 ± 0.1*
		Epirubicin (D)			
	Plastic + No drug	Plastic	Tfd-ECM	cd-ECM	Combi-ECM
p-ERK 1,2	1	0.3 ± 0.2*	0.7 ± 0.1*	0.7 ± 0.2*	1.2 ± 0.1*
p-Akt	1	0.7 ± 0.1*	1.2 ± 0.1*	1.4 ± 0.1*	1.5 ± 0.1*

* $p < 0.05$.

Supplemental Table S7. Relative densitometric quantification of immunoblot band intensities of at least 2 immunoblot gels as illustrated in Figure 9B for the ratio of protein normalized to GAPDH levels. Statistical significance refers relative to either Plastic or Plastic and No drug which is taken as 1. Data is shown as mean ± S.D.

	Cisplatin (top panel)			
	Plastic + No Drug	tfd-ECM	tfd-ECM^{-COL}	tfd-ECM^{-FN}
Bcl-2	1	0.8 ± 0.2	0.4 ± 0.2*	0.2 ± 0.0*
Bcl-xL	1	0.7 ± 0.1*	0.2 ± 0.1*	0.1 ± 0.0*
	Cisplatin (middle panel)			
	Plastic + No Drug	cd-ECM	cd-ECM^{-COL}	cd-ECM^{-FN}
Bcl-2	1	0.8 ±0.2	0.3 ± 0.1*	0.3 ± 0.0*
Bcl-xL	1	0.6 ± 0.2*	0.5 ± 0.1*	0.4 ± 0.1*
	Cisplatin (lower panel)			
	Plastic + No Drug	combi-ECM	combi-ECM^{-COL}	Combi-ECM^{-FN}
Bcl-2	1	0.9 ± 0.2	0.4 ± 0.1*	0.3 ± 0.0*
Bcl-xL	1	0.5 ± 0.2*	0.3 ± 0.0*	0.4 ± 0.1*

* $p < 0.05$.

Supplemental Table S8. Oligonucleotide primer sequences used for qRT-PCR

Gene	Forward	Reverse
GAPDH	5-GCTCTCCAGAACATCATCC-3′	5′-GCCTGCTTCACCACCTTC-3′
BCL-2	5′-CTGCACCTGACGCCCTTCACC-3′	5′-CACATGACCCCACCGAACTCAAAGA-3′
BCL-xL	5′-GATCCCCATGGCAGCAGTAAAGCAAG-3′	5′-CCCCATCCCGGAAGAGTTCATTCACT-3′
MMP1	5′-GCTGGGAGCAAACACATCTGACCT-3′	5′-TGAGCCGCAACACGATGTAAGTTG-3′
MMP-2	5′-CCG CCT TTA ACT GGA GCA AA-3′	5′-TTT GGT TCT CCA GCT TCA GG-3′
MMP3	5′-CTGGGCCAGGGATTAATGGAG-3′	5′-GCTTCAGTGTTGGCTGAGTG-3′
MMP-9	5′- GAG ACA GCA TGG CCA AAT TA -3′	5′- CTC TAG AAA CTG CTG AGG GC-3″
COL1A1	5′-GATTGAGACCCTTCTTACTCCTGAA-3′	5-′TTTGTATTCAATCACTGTCTTGCC-3′
COL1A2	5′-GATTGAGACCCTTCTTACTCCTGAA-3′	5′-GGGTGGCTGAGTCTCAAGTCA-3′
COL2A1	5′-GTCCCAGGATGAGGTCAAGA-3′	5′-TGGCAAGCTCATTGTAGTCG-3′
COL3A1	5′-AAGGTCCAGCTGGGATACCT-3′	5′-CACCCTTTAATCCAGGAGCA-3′
Fibronectin	5'-AGCAGACCCAGCTTAGAGTT-3'	5'-GCAGAAGTGTTTGGGTGACT-3'
ITGA1	5′-GGTGCTTATTGGTTCTCCGTTAG-3′	5′-TTCTCCTTTACTTCTGTGACATTGG-3′
ITGA2	5′-GACCTATCCACTGCCACATGTGAAAAA-3′	5′-CCACAGAGGACCACATGTGAGAAAA-3′

ITGA3	5′-AAGGGACCTTCAGGTGCA-3′	5′-TGTAGCCGGTGATTTACCAT-3′
ITGA5	5′-TGCAGTGTGAGGCTGTGTACA-3′	5′-GTGGCCACCTGACGCTCT-3′
ITGA6	5′-GCCAGCAAGGTGTAGCAGCTA-3′	5′-TTGCTCTACACGAACAATCCCTTT-3′
ITGA11	5′-GGAGGAAGACTTGCGTCG-3′	5′-CACAGGTTCCCCAGTAGATG-3′
ITGB1	5′-GAAGGGTTGCCCTCCAGA-3′	5′-GCTTGAGCTTCTCTGCTGTT-3′
LAMA1	5′-GTCAGCGACTCAGAGTGTTTG-3′	5′-AACTTGGGTGAAAGATCGTCAG-3′
LAMA2	5′-GAACCCGCAGTGTCGAATCT-3′	5′-GGGGAGTTAGCTGCCTTCA-3′
LAMA3	5′-TAGACTTTGGAAGCACCTACTCA-3′	5′-GTTTATCAAGGACACCACAACCT-3′

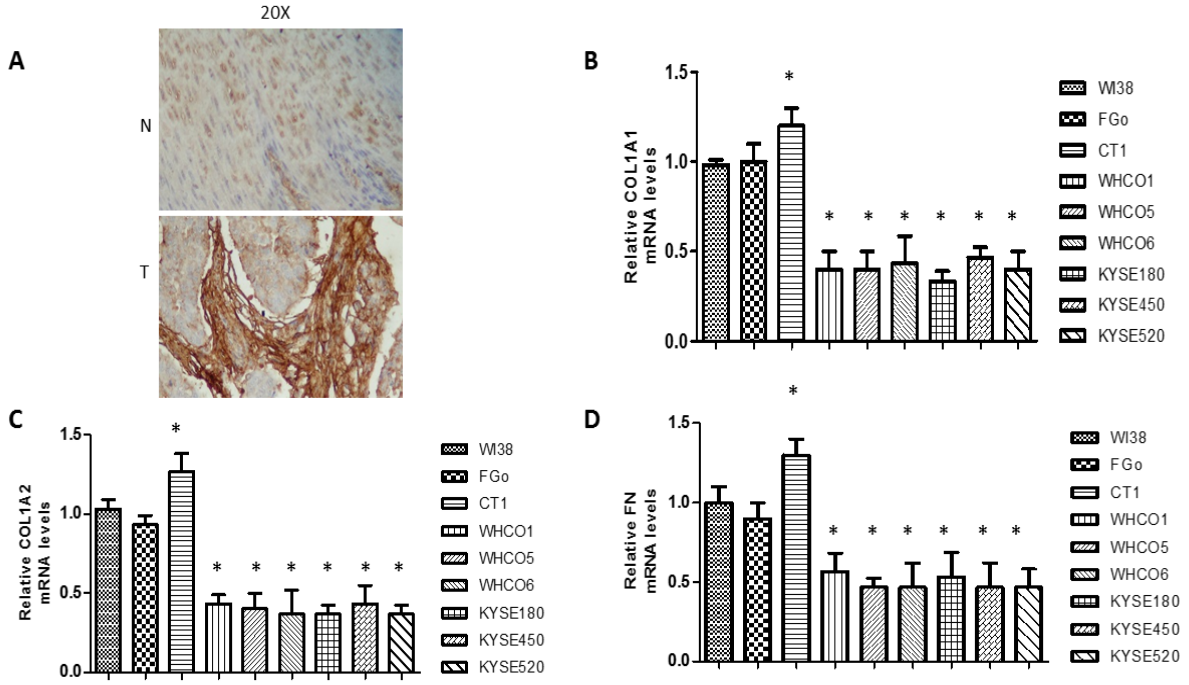

Supplemental Figure S1 Type I collagen and other ECM proteins expression in human ESCC specimens and cell lines (A) Representative Immunohistochemical staining of type I collagen in ESCC specimens versus corresponding normal specimens (B-D) Expression of COL1A1, COL1A2 and Fibronectin in several fibroblasts (WI38, FG_0 and CT1) and ESCC cell lines (WHCO1, WHCO5, WHCO6, KYSE 180, KYSE 450 and KYSE 520). GAPDH was used as a normaliser. * $p < 0.05$.

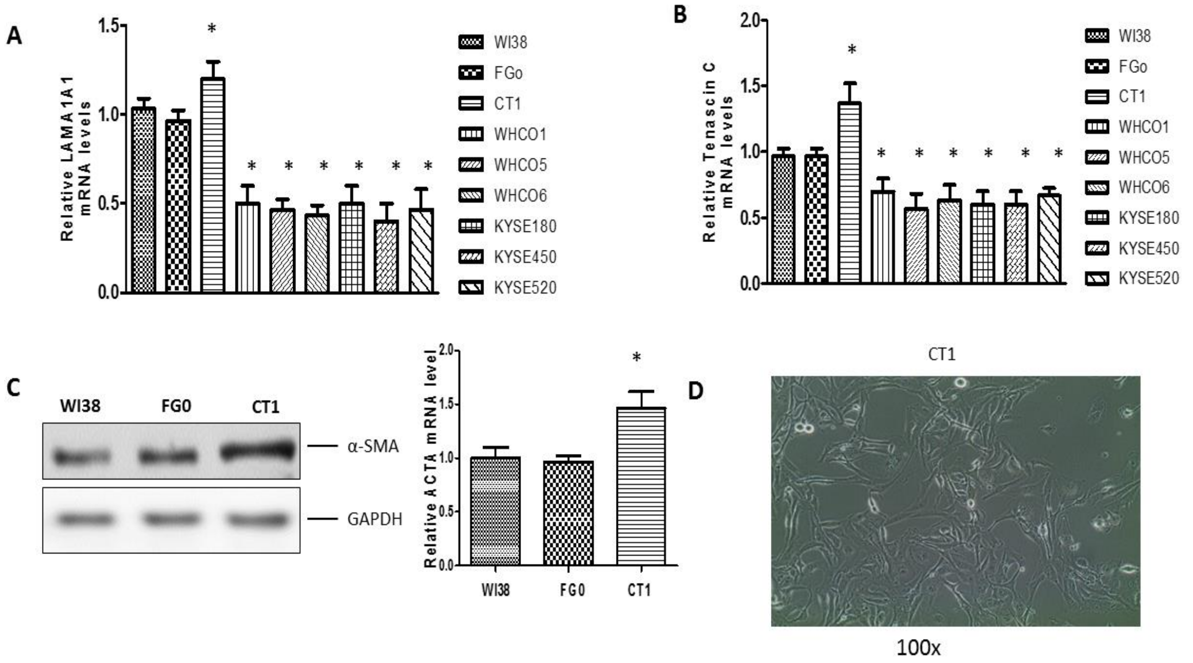

Supplemental Figure S2 (A-B) Expression of LAMA1A1 and Tenascin C in several fibroblasts (WI38, FG$_0$ and CT1) and ESCC cell lines (WHCO1, WHCO5, WHCO6, KYSE 180, KYSE 450 and KYSE 520). RT-PCR was performed using GAPDH as a normaliser. * $p < 0.05$. (C) CT1 fibroblasts express increased levels of α-smooth muscle actin, a marker of tumour associated fibroblasts, than normal fibroblasts WI38 and FG$_0$. (D) Morphology of CT1 fibroblasts. Representative phase contrast image of CT1 fibroblasts.

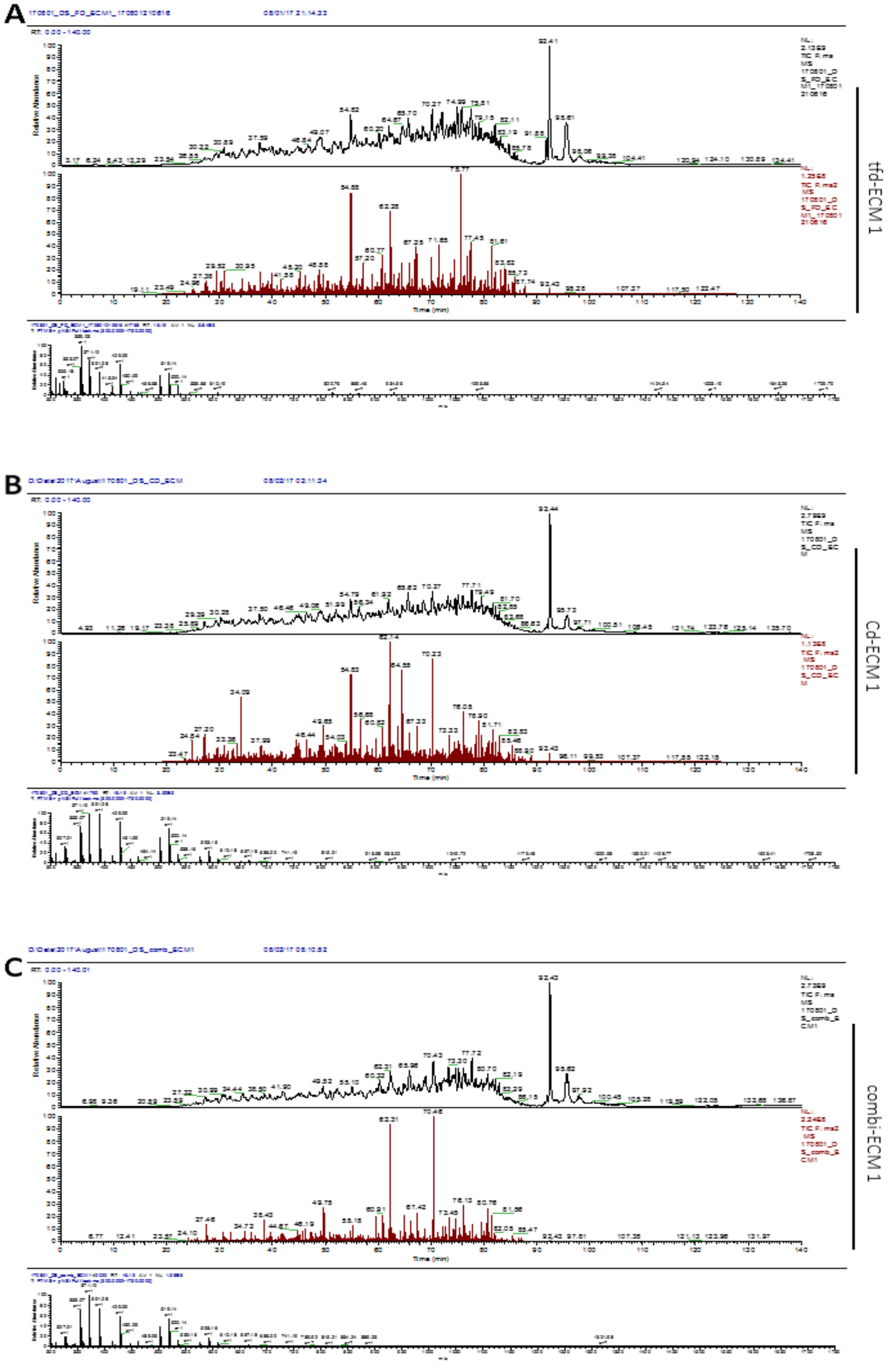

Supplemental Figure S3 Representative mass spectrometric chromatograms of decellularised ECMs (A) Transformed fibroblasts-derived ECM (B) Cancer cell-derived ECM (C) Combinatorial-derived ECM

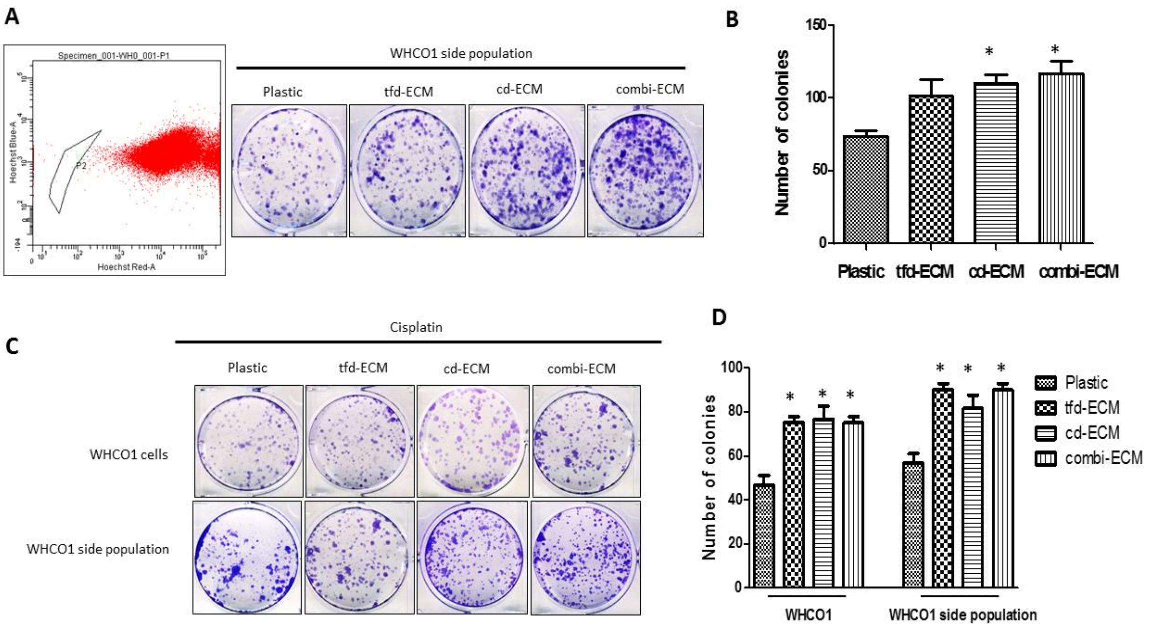

Supplemental Figure S4 Decellularised ECMs promote WHCO1 cancer stem cell colony formation (A) Representative images showing WHCO1 side population sorting and the resulting colonies obtained. (B) Quantification of colony formation from (A). (C) Representative images showing colonies obtained from WHCO1 cells and WHCO1 side population in the presence of cisplatin (D) Quantification of colony formation from (C). * $p < 0.05$.

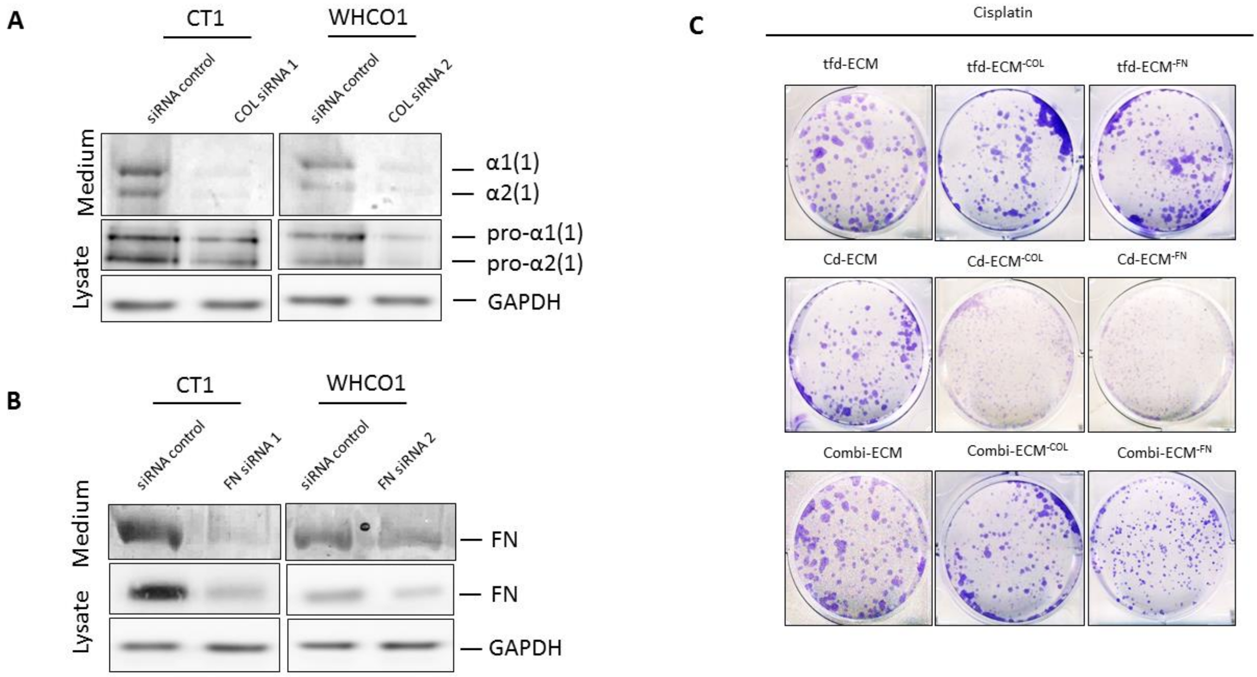

Supplemental Figure S5 (A-B) Knockdown of type I collagen and Fibronectin in CT1 fibroblasts and WHCO1 cells during ECM synthesis was confirmed by SDS-PAGE and immunoblot analysis using medium samples and cell lysates respectively. (C) Representative images of WHCO1 colonies formed when WHCO1 cells are cultured on collagen- and fibronectin-deficient ECMs in the presence of cisplatin.

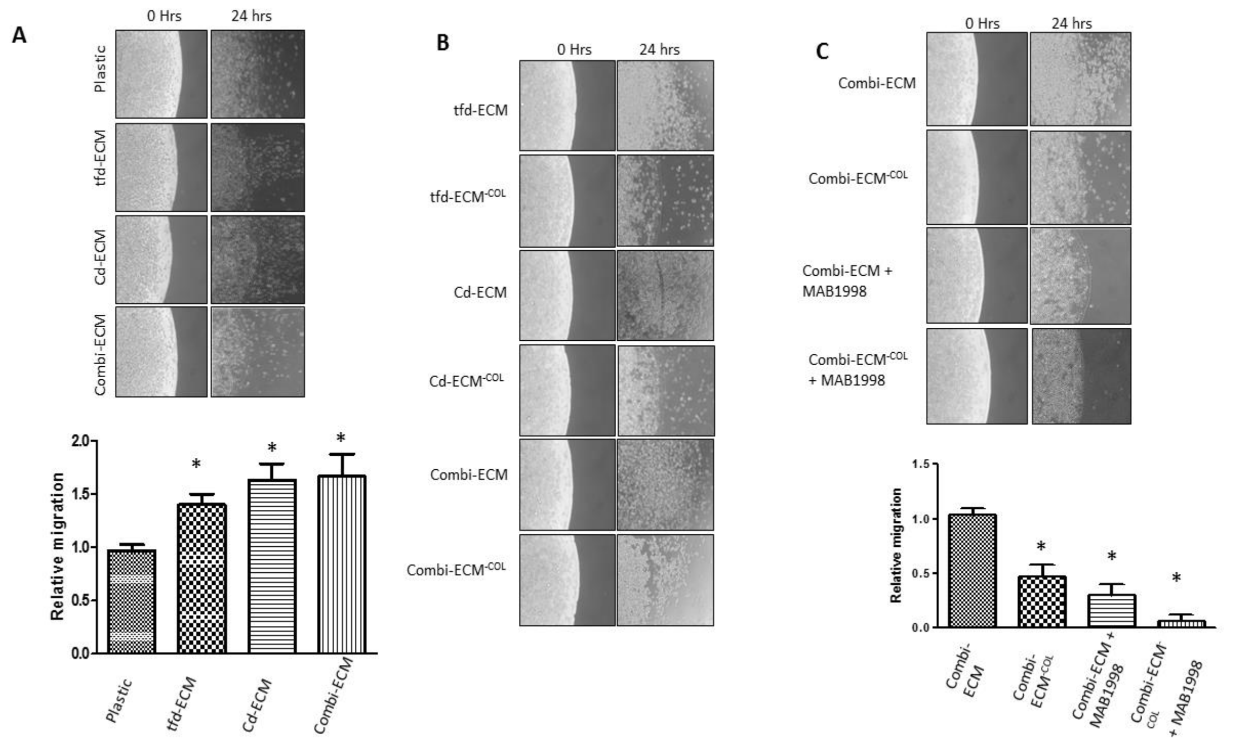

Supplemental Figure S6 Collagen-deficient ECMs significantly reduces WHCO1 cell migration (A) Decellularised ECMs promote WHCO1 cancer cell migration. Representative images of migrating cells (top panel) and quantification of distance migrated (lower panel) (B) Collagen-deficient decellularised ECMs significantly reduce WHCO1 cancer cell migration. Representative images of migrating cells after culturing WHCO1 cells on normal ECMs and collagen-deficient ECMs for 24 hrs (C) Collagen knockdown in combinatorial ECMs and $\alpha 2$ integrin-blocking synergistically reduced WHCO1 cancer cell migration (top panel). Representative images of migrating cells after culturing WHCO1 cells on combi-ECM, collagen-deficient combi-ECMs and combined combi-ECM and incubation with MAB1998 antibody. Quantification of distance migrated (lower panel). * $p < 0.05$.
